# Metabolomics and Microbiomics Insights into Differential Surface Fouling of Three Macroalgal Species of *Fucus* (Fucales, Phaeophyceae) That Co-Exist in the German Baltic Sea

**DOI:** 10.3390/md21110595

**Published:** 2023-11-16

**Authors:** Ernest Oppong-Danquah, Martina Blümel, Deniz Tasdemir

**Affiliations:** 1 GEOMAR Centre for Marine Biotechnology (GEOMAR-Biotech), Research Unit Marine Natural Products Chemistry, GEOMAR Helmholtz Centre for Ocean Research Kiel, Wischhofstrasse 1–3, 24148 Kiel, Germany; eoppong-danquah@geomar.de (E.O.-D.); mbluemel@geomar.de (M.B.); 2 Faculty of Mathematics and Natural Science, Kiel University, Christian-Albrechts-Platz 4, 24118 Kiel, Germany

**Keywords:** seaweed, *Fucus*, surface fouling, molecular networking, untargeted metabolomics, microbiome, NGS, amplicon sequencing

## Abstract

The brown algal genus *Fucus* provides essential ecosystem services crucial for marine environments. Macroalgae (seaweeds) release dissolved organic matter, hence, are under strong settlement pressure from micro- and macrofoulers. Seaweeds are able to control surface epibionts directly by releasing antimicrobial compounds onto their surfaces, and indirectly by recruiting beneficial microorganisms that produce antimicrobial/antifouling metabolites. In the Kiel Fjord, in the German Baltic Sea, three distinct *Fucus* species coexist: *F. vesiculosus*, *F. serratus*, and *F. distichus* subsp. *evanescens*. Despite sharing the same habitat, they show varying fouling levels; *F. distichus* subsp. *evanescens* is the least fouled, while *F. vesiculosus* is the most fouled. The present study explored the surface metabolomes and epiphytic microbiota of these three *Fucus* spp., aiming to uncover the factors that contribute to the differences in the fouling intensity on their surfaces. Towards this aim, algal surface metabolomes were analyzed using comparative untargeted LC-MS/MS-based metabolomics, to identify the marker metabolites influencing surface fouling. Their epiphytic microbial communities were also comparatively characterized using high-throughput amplicon sequencing, to pinpoint the differences in the surface microbiomes of the algae. Our results show that the surface of the least fouling species, *F. distichus* subsp. *evanescens*, is enriched with bioactive compounds, such as betaine lipids MGTA, 4-pyridoxic acid, and ulvaline, which are absent from the other species. Additionally, it exhibits a high abundance of the fungal genera *Mucor* and *Alternaria*, along with the bacterial genus *Yoonia-Loktanella*. These taxa are known for producing antimicrobial/antifouling compounds, suggesting their potential role in the observed fouling resistance on the surface of the *F. distichus* subsp. *evanescens* compared to *F. serratus* and *F. vesiculosus*. These findings provide valuable clues on the differential surface fouling intensity of *Fucus* spp., and their importance in marine chemical defense and fouling dynamics.

## 1. Introduction

The *Fucus* spp. (wracks) represent a keystone species in the littoral zone of rocky-shore ecosystems around the world. Three *Fucus* spp., namely, the *F. vesiculosus* Linnaeus, 1753 (bladder wrack), *F. serratus* Linnaeus, 1753 (toothed or serrated wrack), and *F. distichus* subsp. *evanescens* (C. Agardh) H. T. Powell, 1957 (two-headed wrack), co-occur on the hard substrates in the Kiel Fjord, in the German Baltic Sea (www.marinespecies.org, accessed on 6 October 2023). These three foundation species, collectively, constitute the *Fucus* belt, and create extensive intertidal and subtidal habitats that provide nursery grounds for numerous species. Their presence substantially contributes to shaping and maintaining the overall structure and health of coastal ecosystems. They play pivotal roles in primary production and nutrient cycling, and offer a wide range of essential ecosystem services [1,2]. *Fucus vesiculosus* and *F. serratus* are native to the Kiel Fjord, and are widely distributed along the Atlantic coasts of Europe and North America [3], while *F. distichus* subsp. *evanescens* is an invasive species accidentally introduced from the Arctic region in the late 20th century [4,5]. Similar to any other natural or man-made surfaces submerged in seawater, the thalli of algae are prone to fouling, i.e., colonization by other organisms (epibionts). Microorganisms, especially bacteria, are often the primary macroalgal epibionts [6]. The bacteria secrete exopolymers, which (ir)reversibly attach them to the surface (substrata), with their subsequent aggregation leading to a thin bacterial biofilm [7,8]. This biofilm forms the basis for the further settlement of other microfoulers, such as protozoans, flagellates, fungi, and macrofoulers, e.g., bryozoans or barnacles [9]. Prominent among the macrofoulers on the *Fucus* species are the epizoans *Electra pilosa*, *Mytilus* spp., and *Amphibalanus improvises* [10].

Macroalgae have evolved innate physical and chemical defense mechanisms to control epibiosis. The physical defense mechanisms include the shedding of surface tissue (epithallus sloughing), as observed in rhodolith-forming algal species, such as *Hidrolithon* and *Neogoniolithon* [11]. The chemical defense of the macroalgal host involves the production and release of oxygen radicals (oxidative bursts) in response to chemical signals from epibionts on the surface, as well as cell wall breaches [12]. Furthermore, macroalgae produce diverse metabolites and release them onto their surfaces to actively shape their epibiomes and modulate the settlement of colonizers [13,14]. One example of such a metabolite is brominated heptanone, produced and stored in the surface cells of the red alga *Bonnemaisonia hamifera*, which serves as an antifouling agent [15].

Macroalgae release dissolved organic matter and chemicals, such as sugars and amino acids, to attract beneficial microbes onto their surfaces, thereby increasing the selectivity of epibiosis [16,17]. In return, some epibionts functionally modulate the performance, elasticity, and health of the algal host by supplying essential nutrients and metabolites [7]. As an example, the bacterium *Mesorhizobium loti* supplies vitamin B12 to the green alga *Lobomonas rostrata* [18,19], while other epibionts release bioactive compounds that prevent the settlement of pathogens and other colonizers [20]. On the contrary, some epibionts may facilitate the further colonization of other prokaryotes and eukaryotes, or even increase the susceptibility of the host to grazers [21,22]. For example, the biofilm-forming *Vibrio anguillarum* enhances the settlement of *Ulva* zoospores, while the bryozoan *Membranipora membranacea* on the red seaweed *Cryptonemia seminervis* alters its susceptibility to sea urchin and amphipod grazing [16,23]. These examples illustrate the complex and dynamic nature of the interactions between the seaweed host and its epibiome, largely defined on the host surface. Extensive epibiosis may lead to increased weight and surface roughness, consequently increasing the deposition of particulate materials onto the algal host surface [10,17]. This further reduces surface irradiance and oxygen intake, leading to poor photosynthesis, stunted growth, and eventually promotes algal diseases. Like all macroalgae, the *Fucus* spp. also have the capacity to modulate their surface epibionts. The carotenoid fucoxanthin and polyphenolic phlorotannins are some examples of the known metabolites released onto the surfaces of the *Fucus* spp. to control fouling [24,25].

In a previous study, we analyzed the surface epibiome and the spatial distribution of the surface metabolome of the Kiel Fjord *F. vesiculosus* using LC-MS/MS and Desorption Electrospray Ionization Imaging Mass Spectrometry (DESI-IMS) [26]. Following this initial study, we observed that *F. vesiculosus* was more intensely fouled compared to the other two co-occurring *Fucus* spp., *F. serratus* (less fouled) and *F. distichus* subsp. *evanescens* (the least fouled). Earlier reports have further shown that the epibacterial community of the *Fucus* spp. differs qualitatively and quantitatively among species in the different levels of the intertidal zone [27,28,29,30]. This led us to hypothesize that the associated surface microbiota and metabolome may be the potential drivers of the differential fouling intensity on the surfaces of these three co-occurring *Fucus* spp. We first employed an untargeted metabolomics approach using the Feature-Based Molecular Networking (FBMN) [31] tool, to comparatively analyze the algal surface metabolomes. We also comparatively characterized the prokaryotic and eukaryotic communities associated with the thallus surface of the *Fucus* spp. using amplicon sequencing. Multivariate analyses, including a linear discriminant analysis, allowed us to infer the significant markers driving the chemical and microbial differences on the surfaces of the *Fucus* spp. While existing research has mainly explored the roles of antimicrobial metabolites and epibiont communities separately, our work takes a comprehensive, multidisciplinary approach to comparatively evaluate the co-occurring *Fucus* spp. to advance our understanding of their chemical and microbial ecology.

## 2. Results

### 2.1. Macroscopic Fouling Intensity

**Figure 1:**
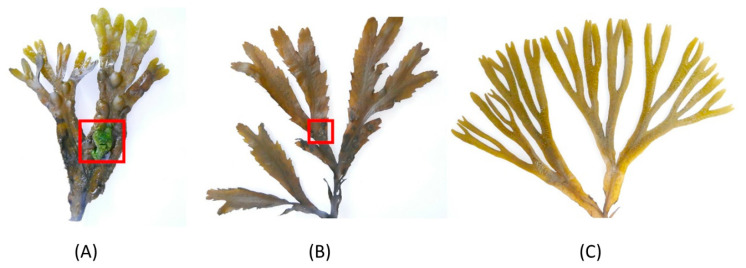
Macroscopic images of the thalli of three *Fucus* species sampled from Kiel Fjord. (**A**) The most intensely fouled *F. vesiculosus* (FV) included macrofouling, as highlighted by the red square, (**B**) the less fouled *F. serratus* (FS) had a small fouled area marked by the red square, and (**C**) the least fouled *F. distichus* subsp. *evanescens* (FE).

Prior to cleaning with artificial seawater, representative algal individuals were visually inspected to confirm our differentially fouled observation. They were assessed using the direct visual enumeration/size of macrofoulers on one thallus branch. As shown in Figure 1, different fouling intensities were observed visually on the different species; the thalli of *F. vesiculosus* were the most intensely fouled, *F. serratus* appeared to be relatively less fouled, and *F. distichus* subsp. *evanescens* was the cleanest (the least fouled).

### 2.2. Comparative Untargeted Metabolomics of Crude Extracts

All the extracts were analyzed using ultra-high-resolution mass spectrometry (UPLC-MS) in both the positive (+) and negative (−) ion modes. The manual inspection of the acquired UPLC-MS chromatograms revealed more complex profiles in the positive ion mode than in the negative ion mode. The acquired UPLC-MS (+) chromatograms revealed fewer peaks in the solid-phase (C18) adsorption surface (SA) extracts (Figure S1) compared to the solvent dipping surface (SD) extracts (Figure S2) of all three *Fucus* spp. Also, the metabolite compositions of the surface-free extracts (SFA, Figure S3 and SFD, Figure S4) were similar to the whole algal extracts (W, Figure S5).

#### Comparison of the Surface Metabolomes of SA and SD

**Figure 2:**
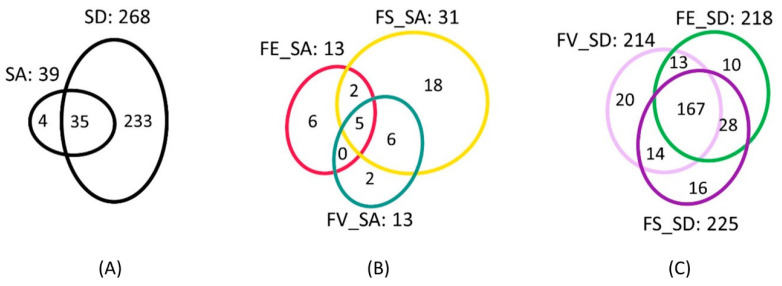
Venn diagrams displaying the distribution of features between (**A**) surface adsorption (SA) and solvent dipping (SD) methods (**B**) for *F. vesiculosus* (FV), *F. serratus* (FS), and *F. distichus* subsp. *evanescens* (FE) using SA, and (**C**) FV, FS, and FE using SD.

The surface extracts represented the focus of our analyses, as their constituents are considered to have the largest impact on epibiosis and fouling on the algal surface. Two surface extraction protocols revealed a vast difference in the number of observed metabolites. The SD method produced 268 features, while the SA generated 39 features (Figure 2A). SA shared 90% of its features (35) with SD, and had only four unique features, putatively annotated to 4-pyridoxic acid, a sphingolipid, and two unannotated ions (Table S1). The SD extracts contained 233 unique features (Figure 2A). The feature distributions among the species, based on the surface extraction techniques, are displayed in Figure 2B,C. *Fucus serratus* showed the highest number of features in both extraction methods.

In order to facilitate the annotation of significant features and the surface metabolomes, Feature-Based Molecular Networking (FBMN) via the GNPS platform [26] was performed to organize the UPLC-(+)-MS/MS data about the extracts into clustered ions with similar MS/MS spectra. An extra network, which used the data obtained in the negative ion mode, was also generated (Figure S6). Only the annotations manually confirmed by the molecular formula prediction using MassLynx^®^ software (v4.2, Waters^®^, Milford, MA, USA), fragment verification, and the source of the hit compound are displayed in the MNs.

**Figure 3:**
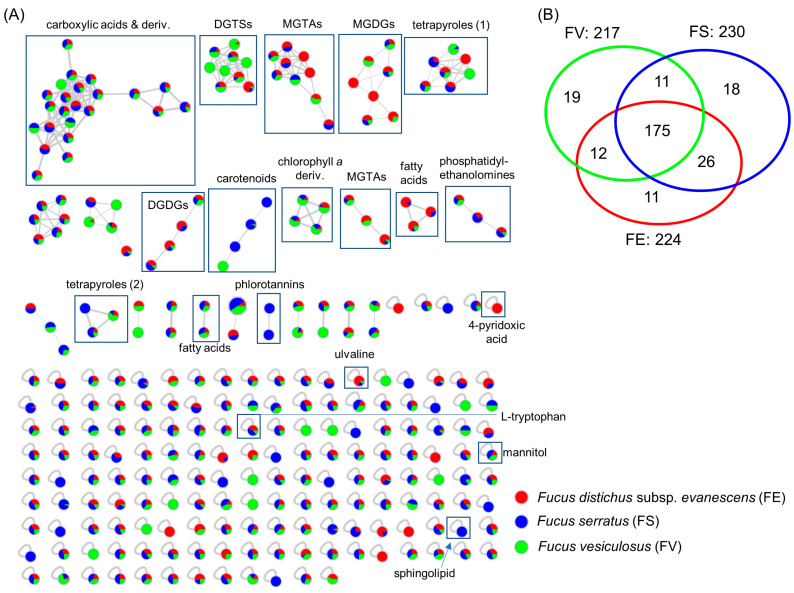
(**A**) MN generated from UPLC-(+)-ESI-MS/MS data of all algal surface extracts. Node sizes are modulated according to the sum of intensities of the ions in all the extracts, while the colors in the pie chart of each node represent the relative quantity of the ion from FE (red), FS (blue), and FV (green). (**B**) Venn diagram displaying the nodes distribution among the species from the combined surface extracts. Betaine lipids are diacylglyceryl trimethyl-homoserine (DGTS) and monoacylglyceryl hydroxymethyl-trimethyl-β-alanine (MGTA). Galactolipids are monogalactosyl diacylglycerol (MGDG) and digalactosyl diacylglycerol (DGDG).

The MN of the surface metabolomes comprised 272 nodes in 24 clusters (Figure 3A). The node size, a reflection of the sum of precursor intensity, allowed for the visualization of the most abundant compounds within the clusters, while the pie chart for each node shows the relative abundance of the metabolite in each *Fucus* sp. The largest cluster of the surface metabolome displayed defined product ions either at *m*/*z* 212.2401 or *m*/*z* 240.2682 in all 3 spp. However, manual dereplication did not provide any structural information and, hence, the annotation of individual ions was not possible. The MolNetEnhancer with the in silico tool NAP annotated the cluster as ‘carboxylic acid and derivative’ class of compounds (Figure 3A and Table S1). Polar lipids, such as betaine lipids and galactolipids, were putatively identified as dominant metabolites across the surfaces of the *Fucus* spp. These include the betaine lipids, diacylglyceryl trimethyl-homoserine (DGTS) and monoacylglyceryl hydroxymethyl-trimethyl-β-alanine (MGTA), and the galactolipids, monogalactosyl diacylglycerol (MGDG) and digalactosyl diacylglycerol (DGDG) (Figure 3A and Table S1). Also, the tetrapyrrole class of compounds was shared by all three *Fucus* spp. These clusters are chlorophyll-related and prevalent in microalgae residing on seaweed surfaces [27]. Other metabolites putatively identified on the surfaces include the carotenoids, fucoxanthinol, dehydrated fucoxanthin, and other cluster members shared by all species. Some singletons were annotated; the sugar-polyol mannitol, the amino acid *L*-tryptophan, and the betaine-type amino acid ulvaline were also shared by all seaweed species, albeit at different concentrations. In the negative ion mode, we could annotate four nodes in the sulfolipids cluster (SQDG), two nodes in the phosphatidylethanoloamine cluster, and one node in the lyso-phosphatidylinositol cluster (Figure S6), shared by the *Fucus* spp.

As shown in Figure 3B, the surface extracts of *F. serratus* showed the highest number of metabolites (230 nodes), while the surface extracts of *F. distichus* subsp. *evanescens* and *F. vesiculosus* displayed 224 and 217 nodes, respectively. A high proportion of the nodes (80%) were shared, while a total of 48 nodes were unique to the surfaces of each *Fucus* sp. (18 nodes for *F*. *serratus*, 11 for *F. distichus* subsp. *evanescens*, and 19 for *F. vesiculosus* (Figure 3B)). Of these, six metabolites exclusive to the surface of *F. distichus* subsp. *evanescens* were annotated as the betaine lipids MGTA 20:5 and MGTA 18:3, the galactolipid MGDG 36:4, the sugar-polyol 1-O-β-D-glucopyranosyl-D-mannitol, the pyridine 4-pyridoxic acid, and the fatty acid eicosatetraenoic acid derivative. *Fucus vesiculosus* also showed exclusive betaine lipids, including DGTS 32:3, DGTS 32:2, and DGTS 34:4; the galactolipid MGDG (18:4); and the carotenoid fucoxanthin dehydrated (five annotations). Eight compounds exclusive to *F. serratus* surface were annotated, and included: the sphingolipids N-(1,3-dihydroxyoctadecan-2-yl)acetamide and C20-phytosphingosine; the carotenoid fucoxanthinol; the tetrapyrrole phaeophorbide A methyl ester; and three phlorotannins, fucodiphloroethol (A and/or B) and fucodiphlorethol (A or B).

**Figure 4:**
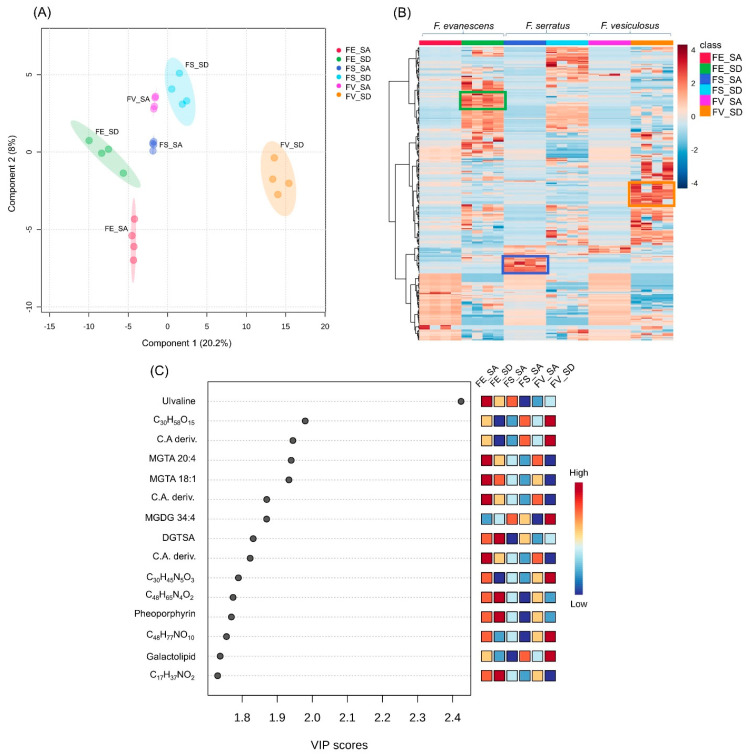
(**A**) PLS-DA scores plot generated from the UPLC-(+)-ESI-MS data of all surface extracts of *Fucus* spp. obtained from C18-adsorption (SA) and solvent dipping (SD) methods. (**B**) Heatmap visualization of the different concentrations of the metabolites, obtained from UPLC-(+)-ESI-MS data, expressed on the surfaces of the three *Fucus* spp. Columns: samples; rows: metabolites; color indicates metabolite expression values, blue—lowest and red—highest. SA and SD extracts for *F. distichus* subsp. *evanescens* (FE_SA/FE_SD), *F. vesiculosus* (FV_SA/FV_SD), and *F. serratus* (FS_SA/FS_SD). Highlighted regions in green, blue, and orange represent area with metabolites in higher abundances. (**C**) Top-15 annotated metabolites ranked by their VIP values. The mini heatmap on the right indicates their concentrations on the surface extracts of the *Fucus* spp.

Next, we performed a multivariate analysis, i.e., a supervised, partial least squares discriminatory analysis (PLS-DA) in order to determine the variations and identify the potential marker compounds in the surface metabolomes. The PLS-DA revealed a clear discrimination among the surface extracts, indicating a total variance of 28.2% (Figure 4A; permutation test result, *p* < 0.05). A hierarchical clustering heatmap was created to visualize the different concentrations of the metabolites on the surfaces of the *Fucus* spp. (Figure 4B). Several of the regions, highlighted in green, blue, and orange, represent areas with metabolites in higher abundances relative to the other regions. The most important metabolites responsible for algal surface discrimination are highlighted with their variable importance in projection (VIP) scores (Figure 4C). These were the betaine lipids, MGTA 20:4, MGTA 18:1, and DGTSA, and ulvaline, which were most abundant on the surface of *F. distichus* subsp. *evanescens*, while the galactolipid MGDG 34:4 was most abundant on the *F. vesiculosus* surface. The carboxylic acid derivative (C.A. deriv.) class of compounds were also significant on *F. vesiculosus* (SD) and *F. distichus* subsp. *evanescens* (SA) (Figure 4C and Figures S7–S10).

Comparing the surface extracts (SA and SD) to the whole (W) and to the surface-free extracts (SFA and SFD), we observed a clear separation/difference between the surface metabolites (SA and SD) and the algal inner-tissue metabolites (W, SFA, and SFD) (Figure S11). Based on the MN (Figure S12), the primary distinction observed between the surface and inner-tissue metabolomes pertained to the unannotated ‘carboxylic acid and derivatives’ cluster. This cluster was exclusively identified in the surface extracts of all the *Fucus* spp., suggesting its origin on the surface. All the annotations made are displayed in Table S1.

### 2.3. Microbiome Analysis

The surface microbiomes of the three *Fucus* spp. were comparatively analyzed using amplicon sequencing of the V3-V4 hypervariable region of the prokaryotic 16S rRNA gene and the eukaryotic internal transcribed spacer 2 region (ITS-2), in order to determine if the epiphytic communities were species-specific, and could illuminate differential fouling levels. Seawater (SW) and the biofilm from a stone (BF) taken from within the *Fucus* meadow were included as references for comparisons.

#### 2.3.1. Epiphytic Bacterial Microbiome

The Illumina NovaSeq sequencing platform generated a total of 7,193,202 raw paired-end reads out of 66 samples (the *Fucus* surface samples and controls, incl. replicates) in total. These were reduced to 2,096,951 bacterial reads after quality filtering, and chimera and chloroplast sequence removal. A rarefaction analysis indicated sufficient sequencing depth (Figure S13). The highest alpha diversity of all the algal species was observed in *F. vesiculosus*, followed by *F. distichus* subsp. *evanescens*, and the lowest alpha diversity was in *F. serratus* (Figure S14). Significantly distinct bacterial alpha diversity was observed (Wilcoxon rank sum test) between the pairs of *F. vesiculosus* and *F*. *serratus* and *F. distichus* subsp. *evanescens* and *F*. *serratus*, but not between *F. vesiculosus* and *F. distichus* subsp. *evanescens* or between the reference samples SW and BF. Moreover, highly significant variations in alpha diversity were observed for *F. serratus* and *F. distichus* subsp. *evanescens* with respect to the BF and SW, whereas *F. vesiculosus* showed only slight (to the BF) or no (to the SW) significant differences. No significant alterations were observed between the different individuals (Figure S15).

**Figure 5:**
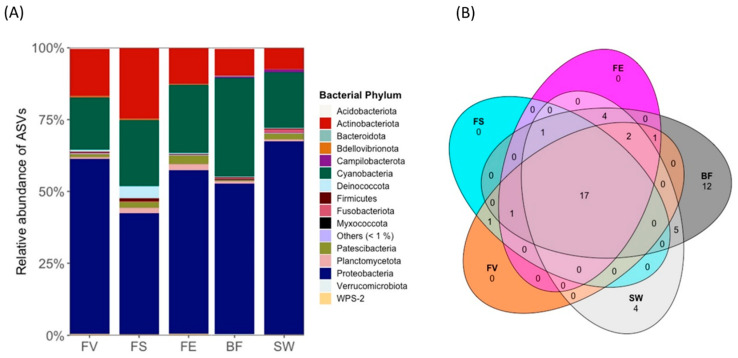
(**A**) Bacterial phyla associated with the surfaces of *Fucus* spp. and stone biofilm (BF) and seawater (SW) references. FV: *Fucus vesiculosus*; FS: *F. serratus*; FE: *F. distichus* subsp. *evanescens.* Others (<1%) represent several phyla with less than 1% relative abundance. (**B**) Venn diagram of bacterial amplicon sequences at genus level.

In total, 15 bacterial phyla (with an abundance > 1%) were observed in the samples (Figure 5A). The epibionts of all three *Fucus* spp. and the reference samples were dominated by three bacterial phyla, Proteobacteria, Cyanobacteria, and Actinobacteria, which constituted 89.8–96.2% of the community in all the samples. The highest abundance of Proteobacteria was in the SW (67.3%), and the lowest on the surface of *F. serratus* (42.1%). The highest Cyanobacteria abundance was detected in the BF (34.5%), and the lowest on *F. vesiculosus* (18.2%). Actinobacteria were most abundant on *F. serratus* (24.6%). Surprisingly, the phylum Bacteroidota was only detected as a rather minor constituent of the bacterial community in all the samples, with the highest abundance on *F. distichus* subsp. *evanescens* (0.12%). The extremophilic bacterial phylum Deinococcota was specific to the seaweeds, with the highest abundance on *F. serratus* (4.2%). The dominant genus on the surfaces of the seaweeds was *Schizothrix* (Cyanobacteria), with 24.41% on *F. vesiculosus*, 29.61% on *F*. *serratus*, and 26.02% on *F. distichus* subsp. *evanescens* (Table S2).

The references SW and BF showed bacterial community differences from the algal surfaces. The phyla Acidobacteriota and Firmicutes were exclusive to the BF and SW. Moreover, *Clade Ia* (Proteobacteria) and *Phormidesmis* (Cyanobacteria) represented the most abundant genera in the SW (22.46%) and BF (12.76%), respectively (Table S2). As shown in the Venn diagram (Figure 5B), 17 genera were identified in all the samples, and no genus was specific to any of the algal surfaces. A beta diversity analysis using Bray–Curtis distances highlighted the dissimilarity of the bacterial communities on each seaweed surface and reference sample (Figure S16, Table S3). The distribution of the bacterial order for the *Fucus* spp. is given in Figure S17.

In order to determine the taxa most likely responsible for the observed variations, a linear discriminant analysis effect size (LEfSe) was performed using the LefSe function implemented in the R microeco package (v. 0.13.0), using a threshold value of four.

**Figure 6:**
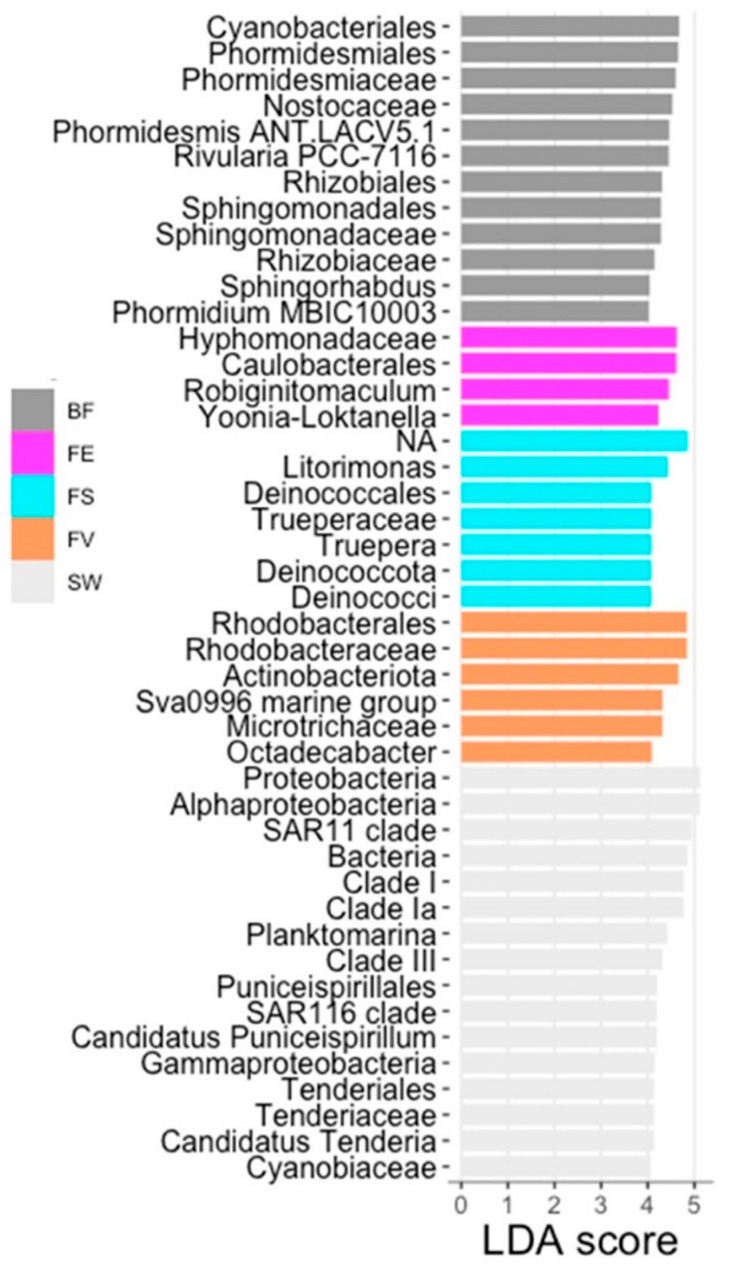
Linear discriminant analysis of bacterial taxa of *Fucus* spp. Bar plots showing linear discriminant analysis (LDA, threshold: 4) score after differential abundance analysis of bacterial taxa using Lefse, according to origin. FV: *Fucus vesiculosus*; FS: *F. serratus*; FE: *F. distichus* subsp. *evanescens*, BF: biofilm on stone; SW: seawater.

This identified three biomarkers for *F. distichus* subsp. *evanescens*, seven for *F*. *serratus*, and six for *F. vesiculosus*, as the taxa responsible for the differences in the three *Fucus* surface microbiomes. The significantly abundant epiphytic bacteria (biomarkers) on *F. distichus* subsp. *evanescens* were the genus *Robiginitomaculum* (Family: Hyphomonadaceae, Order: Caulobacterales, Phylum: Pseudomonadota) and genus *Yoonia-Loktanella* (Phylum: Pseudomonadota). The most significant discriminant genera on the surface of *F. serratus* were *Truepera* (Family: Trueperaceae, Order: Trueperales, Phylum: Deinococcota), *Litorimonas* (Phylum: Pseudomonadota), and a member of the order Deinococcales (Class: Deinococci, Phylum: Deinococcota). Octadecabacter (Family: Roseobacteraceae, Order: Rhodobacterales, Phylum: Pseudomonadota), Microtrichaceae (Phylum: Actinobacteriota), and the Sva0996 marine group (Phylum: Actinomycetota) were the significant discriminant taxa on the surface of *F. vesiculosus* (Figure 6). For the controls, 12 and 16 biomarkers were identified in the BF and SW, respectively (Figure 6).

#### 2.3.2. Comparative Analysis of Eukaryotic Epiphytes

For the eukaryotic community, a total of 6,824,917 raw reads were obtained from 60 samples, as 6 samples (4 BF, 1 SW, and 1 *F. distichus* subsp. *evanescens* sample) failed quality checks/filtering. Quality filtering and chimera removal resulted in 6,053,795 reads and 1,962 taxa for phyloseq analysis. A rarefaction analysis also revealed sufficient sequencing depth (Figure S18). An analysis of the alpha diversity using the Shannon index as well as the ASV diversity showed a considerably lower ITS sequence diversity on all the seaweed surfaces compared to the SW and BF (Figure S19). Slightly significant differences were observed between *F. vesiculosus*/*F. serratus* and *F. vesiculosus*/*F. distichus* subsp. *evanescens*, but not between *F. serratus*/*F. distichus* subsp. *evanescens* and the references SW/BF. The ASV diversity was insignificant between individuals (Figure S20).

**Figure 7:**
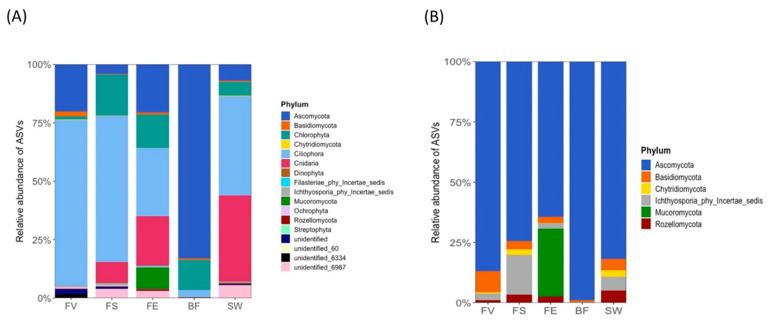
Community composition of epiphytic eukaryotes on *Fucus* spp. based on amplicon sequencing of the ITS region. (**A**) Eukaryotic community composition at phylum level. (**B**) Fungal community composition at phylum level. FV: *Fucus vesiculosus*; FS: *F. serratus*; FE: *F. distichus* subsp. *evanescens*; BF: biofilm on stone; SW: seawater.

The eukaryotic community was dominated by the phyla Ciliophora, Chlorophyta, and Ascomycota on all the algal surfaces (Figure 7). Cnidaria and Ascomycota were more abundant on *F. distichus* subsp. *evanescens* and *F. serratus* than on *F. vesiculosus*, and the relative abundance of Mucoromycota on *F. distichus* subsp. *evanescens* was higher than on *F. serratus* and *F. vesiculosus*. The fungal phylum Ascomycota was, by far, the most abundant phylum in the BF, followed by Chlorophyta. The SW was largely dominated by the phyla Ciliophora and Cnidaria. The relative abundances of the eukaryotes at the genus level are displayed in Table S4. Excepting the BF, fungi accounted for 4–45% of the ITS sequences. A considerably lower frequency of fungi was observed on *F*. *serratus*. On *F. distichus* subsp. *evanescens*, Ascomycota was most abundant, followed by Mucoromycota. Basidiomycota were only detected on the *F. vesiculosus* surfaces in notable abundances. A PERMANOVA analysis of the whole dataset showed significant differences with regard to the sample origin and individuals (Table S5).

With reference to the fungal-derived sequences, the alpha diversity analysis (ASVs) revealed significant differences only between *F. vesiculosus* and *F. serratus*, as well as among the algae compared to the SW and BF (Figures S21 and S22). Ascomycota, Basidiomycota, Ichthyosporia, and Rozellomycota were the most abundant phyla on the algal surfaces (Figure 7B). The *F. distichus* subsp. *evanescens* surfaces exclusively harbored Mucoromycota. Differential abundances of fungi were observed at the order and genus levels (Figure S23, Table S6).

**Figure 8:**
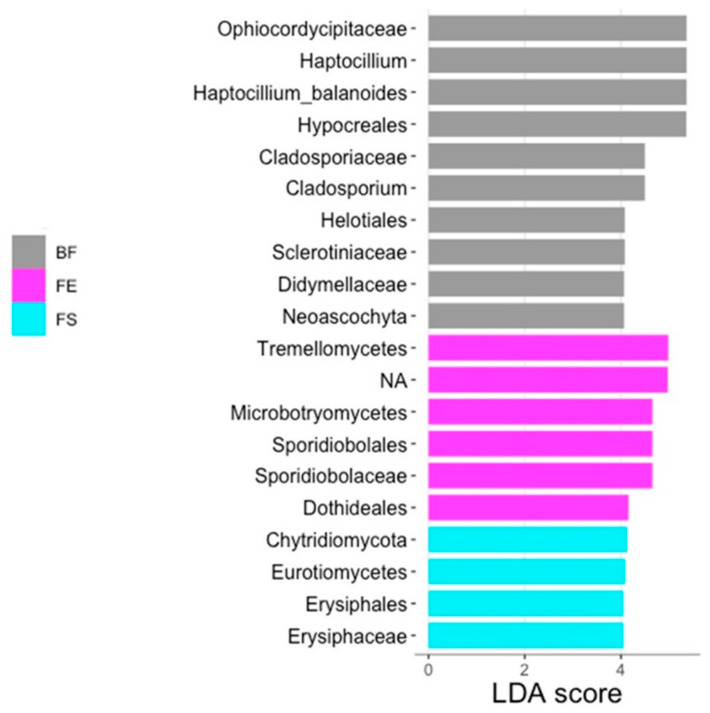
Differential abundance using linear discriminant analysis (LDA, threshold: 4) of fungal taxa based on ITS amplicon sequences, according to sample origin. FV: *Fucus vesiculosus*; FS: *F. serratus*; FE: *F. distichus* subsp. *evanescens*; BF: biofilm on stone; SW: seawater. No differentially abundant taxa were identified for SW or FV.

The most dominant fungal genera on *F. vesiculosus* were *Candida* (82.30%) and *Haptocillium* (9.20%), while *Sphaeroforma* (38.04%) and *Alternaria* (25.00%) were most abundant on *F. serratus* (Table S6). *Mucor* represented the most abundant genus on *F. distichus* subsp. *evanescens* (65.41%), followed by *Alternaria* (14.91%) (Table S6). Due to the reduced sample set (12 samples removed, a total of 189 taxa), a beta diversity analysis and statistical assessment were not successful for the fungal subset. However, an LEfSe analysis identified 10 biomarkers for the BF, 6 for *F. distichus* subsp. *evanescens*, and 4 for *F*. *serratus*. No differentially abundant taxa were identified for the SW or *F. vesiculosus* (Figure 8). Fungi of the orders Sporidiobolales (Division: Basidiomycota) and Dothideales (Division: Ascomycota) were enriched on the surface of *F. distichus* subsp. *evanescens*. On *F*. *serratus*, Erysiphales (Class: Erysiphaceae, Division: Ascomycota), Eurotiomycetes (Division: Ascomycota), and fungi of the division Chydridiomycota were enriched (Figure 8).

## 3. Discussion

There is ample evidence suggesting that seaweeds are able to control and shape their surface epibiome, which is considerably influenced by environmental gradients [28,29,30]. It was, therefore, intriguing to visually observe the differential fouling intensity on the thalli of these three foundation species, *F. distichus* subsp. *evanescens*, *F. serratus*, and *F. vesiculosus*, co-occurring in the Kiel Fjord. In our quest to unravel the different fouling levels on the seaweed surfaces, we thought the composition of the surface metabolome and microbial community were pivotal; hence, the aim of the current study. Therefore, the identification of the unique core taxa of the surface microbiome, and the metabolome associated with the surfaces of the foundation species were of interest. Although the thalli surface metabolomes were the main target of this study, the whole biomass and surface-free algal materials were also extracted and analyzed as controls. The whole algal extracts were analyzed to index their general metabolic profile, while the surface-free algal extracts served to assess the residual metabolome after removal of the surface metabolome. Many known *Fucus*-derived compounds, with reported diverse biological activities, were annotated in all the three species (Table S1).

For the surface extracts, two extraction protocols previously described, surface C18 adsorption (SA) and surface dipping (SD), were employed [31,32,33,34]. Although SA produced fewer compounds (14% of the total surface metabolites), SA extract-specific compounds were annotated, including the sphingolipid *N*-(1,3-dihydroxyoctadecan-2-yl)acetamide [35] and pyridoxic acid, an ROS (reactive oxygen species)-lowering compound [36,37]. Similarly, the surface quorum-sensing metabolite *N*-(3-oxooctanoyl) homoserine lactone was extracted, using SA only (absent from the SD extract), from the surface of *F. vesiculosus* in a previous study [31]. Although this compound was not identified in the current study, the exclusive presence of some compounds in SA supports the complementarity of these two surface extraction techniques.

Our efforts to compare the surface metabolome of the *Fucus* species revealed diverse compounds, as visualized with molecular networks (MN). Based on automated and manual dereplication, about 70% of the extracted compounds were annotated to the class level. Indeed, the surface metabolome of *F. vesiculosus* has been extensively studied, with reported surface metabolites including citric acid, amino acids (*L*-serine, *L*-threonine, *L*-asparagine, and *L*-proline), simple sugars (mannitol, glycitol, and ribitol) and dimethylsulfopropionate [38,39]. In the current study, the amino acid *L*-tryptophan and the sugar mannitol were annotated, with the latter reportedly representing approx. 30% of the dry weight of the *Fucus* spp. [40,41]. Mannitol is an osmoregulatory and energy-storage compound utilized in brown algae in various ways, such as to scavenge oxygen radicals and produce chemical defenses during stress [42,43]. Its production showed a significant negative correlation with microfouling on *F. vesiculosus* [34]. It also displays bacteriostatic activity against multiple *Bacillus* spp. [44]; hence, it contributes to shaping the algal surface microbial community, esp. in *F. distichus* subsp. *evanescens*, which showed a surface-specific mannitol linked to a β-glucopyranose residue (1-O-β-D-glucopyranosyl-D-mannitol) in our study.

Our results show species-specific and shared compounds in significantly different quantities on the *Fucus* spp., which drive the chemical differences on the surfaces. The betaine lipids and galactolipids (lyso-type lipids) MGTA 20:5, MGTA 18:3, and MGDG 36:4, as well as MGTA 20:4 and MGTA 18:1, were identified as discriminant metabolites, mostly abundant or specific on the *F. distichus* subsp. *evanescens* surface. These polar lipids have been associated with diverse bioactivities, including antifouling, antimicrobial, and osmoprotectant [45,46,47,48]. The betaine-type amino acid ulvaline, first isolated from the microalgae *Monostroma nitidum* [49], was observed in significantly higher quantities on *F. distichus* subsp. *evanescens* (FE_SA) than on the other *Fucus* spp. Unfortunately, very little is known about its biological function but, similar to other betaine lipids, it may be relevant to marine algal metabolism and provide antioxidant effects during biotic stress [50,51]. The putative vitamin B12-derivative 4-pyridoxic acid, with antioxidant properties, was also observed only on the surface of *F. distichus* subsp. *evanescens*, and has also been previously identified in some seaweeds as either an exogenous molecule from associated bacteria or self-made [18]. The bioactivities of these *F. distichus* subsp. *evanescens* surface metabolites align with its status as the least fouled surface observed.

On the surface of *F*. *serratus*, we putatively identified three phlorotannins. Phlorotannins are hydrophilic in nature, and form part of the cell wall structures of brown algae, constituting about 5–12% of the dry weight of the *Fucus* spp. [52]. Among their wide range of biological activities, phlorotannins defend algae against grazers [53,54] and may contribute to the reduction in surface fouling of *F*. *serratus*. The antioxidant fucoxanthinol (deacetylated derivative of fucoxanthin [55]) was observed only on the surface of *F*. *serratus*.

Similar to previous work, dehydrated fucoxanthin was observed only on the surface of *F. vesiculosus* [31]. Unlike *F. distichus* subsp. *evanescens*, the surface of *F. vesiculosus* showed more diacyl-betaine lipids, such as DGTS 32:3, DGTS 32:2, and DGTS 34:4. ‘Acyl editing’, the diacylation and reacylation cycles in lipid metabolism [56], could influence their biological activities. However, this remains to be investigated. Other discriminant compounds belonging to the carboxylic acid cluster could not be annotated.

As for the microbiome analysis, the overall prevalence of Proteobacteria, Cyanobacteria, and Actinobacteria in the seawater, stone biofilm, and on the surfaces of the *Fucus* spp. is in line with other studies, as they are usually dominant in marine environments. Proteobacteria are ubiquitous in marine environments due to their metabolic versatility and ability to enhance surface colonization and biofilm formation [57,58]. Our observation of a wide spread of Cyanobacteria on the surfaces of the *Fucus* spp., collected during the summer season, is consistent with previous findings that have shown an abundance of Cyanobacteria on *F. vesiculosus* in summer, but not in winter [59]. Actinobacteria and members of the phyla Planctomycetota, Verrucomicrobiota, and Fusobacteriota, observed in relatively lesser abundances, have previously been reported on many seaweed surfaces [59,60,61]. Notably different from other observations, is the extremely low abundance of the phylum Bacteroidetes in the current study. This may be due to a seasonal effect, as seasonality is a known phenomenon for this phylum [62], and there are studies that have shown that Bacteroidetes decreases significantly towards summer [63].

Compared to the algal surfaces, the stone biofilm (BF) and seawater (SW) generally showed slightly higher bacterial diversity, which further corroborates the findings of multiple studies that have postulated that algae actively selects their associated microbiota. The only bacterial phylum represented on all the algal surfaces, and was significantly abundant on *F*. *serratus*, but not in the SW or BF reference samples, was Deinococcota. Its members are known to be differentially abundant in different geographic regions and on seaweeds, as they play an important role in denitrification and biofilm formation [64,65,66,67]. One member of the phylum is a known producer of the carotenoid deinoxanthin, with known algicidal activity against dinoflagellates [68]; thus, the members of this phylum may have important beneficial effects for seaweed. The alphaproteobacterial genus *Octadecabacter* and members of the actinobacterial Sva0996 marine group were identified as differentially abundant on *F. vesiculosus* using an LefSe analysis, but their ecological roles remain elusive, as does the role of the differentially abundant genus *Robiginitomaculum* on *F. distichus* subsp. *evanescens*. The genus *Yoonia-Loktanella*, also significantly abundant on *F. distichus* subsp. *evanescens*, is known for its ability to utilize complex algal exudates [69]. It is a dimethylsulfopropionate (DMSP) degrader with algicidal potential [70,71]; thus, it may be an important algal associate. Although all three algae were collected from the same spot, their microhabitats differ. *F. vesiculosus* in the Kiel Fjord grows close to the surface, with occasional desiccation events during unfavorable wind conditions, whereas *F. serratus* and *F. distichus* subsp. *evanescens* occur at greater depths. In an earlier report on epibiosis at different seawater depths of 1–6 m, the epibiont mass on *F. vesiculosus* was the lowest, at 6 m [72]. Rohde et al. [72] asserted that the alga had to actively control fouling on its surface to overcome the stress from diminishing light intensity, which increases with increasing depth, in order to avoid the shading effects of the epibionts. This may be true for our current study, as *F. vesiculosus* (shallow) was more fouled than *F. distichus* subsp. *evanescens* and *F. serratus* (deep).

Most published studies have focused on the bacterial epiphytic community. A recent study, however, suggested taking eukaryotic diversity into account for an explanation of prokaryotic community composition and dynamics in aquatic habitats [73]. In this study, ciliates were detected as the most abundant eukaryotes based on ITS analyses, except for the BF reference, where Ascomycota were most abundant. Ciliates play key roles in the microbial food web [74]. They are known bacterial grazers and, hence, generally influence bacterial epibiosis. However, the ciliate genus *Zoothamnium*, which was detected in high abundances on *F. vesiculosus*, is also known as a marine pathogen [75], and may contribute in part to the highly fouled surface observed. The significantly abundant ascomycete genus on *F. vesiculosus* was the yeast *Candida*, which might contribute to microfouling and consume algal exudates. The Ichthyosporea represents a fungus-like protozoan lineage that has not been further classified. Their members, including the genus *Sphaeroforma*, which showed high abundances on the *F. serratus* surface, have been previously detected in marine invertebrates [76], but their role is, as of yet, unclear. *Alternaria*, which was second-most abundant fungal genus on *F. serratus* and *F. distichus* subsp. *evanescens*, but only constituted 1.6% of the fungal community on *F. vesiculosus*, is known as an algal epiphyte known for producing compounds, e.g., terpenes active against the bacterial pathogens of algae [77]. Sporidiobolales was significantly abundant on *F. distichus* subsp. *evanescens* using LEfSe analysis. A member of this taxon is considered an antagonistic yeast with profound biocontrol efficiency against pathogens, and may have a positive relevance on the surface of *F. distichus* subsp. *evanescens* [78]. Although the genus *Mucor* is generally known as endophytic or soil derived, it has been also isolated from red algae [79]. Its main secondary metabolite was identified as tyrosol, which displays antibiofilm activities in medical settings [80] and may exert a similar ecological role on the *F. distichus* subsp. *evanescens* surface. It is noteworthy that surface microbial metabolites must be present in ecologically relevant concentrations to exert a meaningful influence on epiphytic communities. Dose–response relationships are vital, as the effects depend on metabolite concentration, community dynamics, spatial distribution, and temporal changes [81,82]. Studying these concentration–response relationships is essential for understanding the ecological significance of these microbial metabolites.

Unfortunately, not many microbial metabolites were putatively identified in this study, although the surface extracts represent the combined surface metabolome of the *Fucus* spp. and their associated epibionts. In the previous work, we annotated many microbial secondary metabolites in the surface extract of *F. vesiculosus* [31]. This may be due to factors such as seawater temperature, weather, and seasons, which tend to influence the surface metabolomes of seaweeds [83]. In the current study, the unannotated nodes in the MN may represent unidentified microbial compounds, while microbial compounds in minute amounts may have been excluded during data pre-processing.

In addition to the chemical defense mechanisms involving the antimicrobial metabolites produced by the host seaweed and its epiphytes, seaweed surface fouling is profoundly influenced by a multitude of environmental and biological factors. These factors, in turn, contribute to the observed variations in fouling intensity among the different *Fucus* spp., and even within the same species. Water quality parameters, such as temperature, salinity, and nutrient availability (nitrogen, phosphorus, and carbon), significantly impact fouling dynamics [84]. Hydrodynamic forces, mediated by wave action, can either facilitate or hinder fouling, by physically removing or depositing fouling material on individual species [85]. Seasonal variations, geographic location, light, and anthropogenic disturbances further contribute to the complex mosaic of factors shaping fouling on seaweed surfaces [86]. Understanding these multifaceted interactions is critical for unraveling the ecological dynamics of fouling on macroalgae. However, the objective of this study was to comprehend the metabolomic and epiphytic microbial influence on the different fouling levels of the co-occurring *Fucus* spp. As all three *Fucus* spp. were sampled from the same location, abiotic factors, such as water quality, were considered to be identical. All three algal species have a high and comparable polysaccharide composition [87]. Morphologically, they consist of a holdfast, a stipe, and flattened dichotomously branched blades (Figure 1). Unlike the others, *F. vesiculosus* has air-filled vesicles that keep it afloat and may expose it to air-borne settlers. *Fucus distichus* subsp. *evanescens* presents with a smooth surface topography with slender blades. An earlier study reported *F. distichus* subsp. *evanescens* to be less fouled than *F. vesiculosus*, after comparing their biomasses and the variation in their epiphytic community composition [88]. The low epiphytic growth on *F. distichus* subsp. *evanescens* was partly attributed to its morphology, i.e., the smoother surface of its fronds, the thickness of its cell wall, and surface texture [88]. Rickert et al. [42] also reported a lesser seasonal epiphyte recruitment pattern in *F. distichus* subsp. *evanescens* than in *F*. *serratus*. It is, therefore, conceivable that in addition to the surface metabolite and microbiome, the thallus morphology of *F. distichus* subsp. *evanescens* makes it a less suitable substratum for epibiosis and, hence, having the least fouling state observed, relative to the other *Fucus* spp.

## 4. Materials and Methods

### 4.1. Sampling and Sample Processing

Fresh seaweed materials were sampled from Kiel Fjord, in the vicinity of the Bülk lighthouse (54°27′15.6” N and 10°11′55.0” E) in July 2019. Approximately 1.5 kg each of *F. vesiculosus*, *F. distichus* subsp. *evanescens*, and *F. serratus* were collected within a radius of 10 m. *Fucus vesiculosus* was retrieved from depths not exceeding 0.5 m, while *F. distichus* subsp. *evanescens* and *F. serratus* were collected at a maximum depth of 1 m from the water’s surface. Air and water temperatures were 16 °C and 17.5 °C, respectively, and the water salinity was 13.1 PSU with a pH of 7. Following collection, algal materials were placed separately into sterile plastic bags. Ambient seawater (SW) was collected in sterile 1 L Schott bottles. The biofilm on the surface of a stone (BF) close to the algae was also sampled into sterile 50 mL Falcon tubes. All samples were transported to the laboratory in a cooling box and processed on the same day. Algal samples were carefully rinsed with artificial seawater prepared by dissolving 1.8% Instant Ocean^®^ in milliQ water (Arium^®^ Lab water systems, Sartorius) to remove surface debris and loosely attached macrofoulers.

### 4.2. Algal Extractions

Algal surface extractions were carried out using both solvent dipping and solid-phase adsorption methods, as described in Parrot et al. [31]. Briefly, the solvent dipping method was achieved by dipping the algal thalli into a stirring mixture of *n*-hexane:MeOH (1:1) for 4 sec (1 kg algal material in 1 L solvent mixture) to extract, specifically, the surface-associated metabolites without leaching the metabolites present in the inner algal tissues [38,82]. The resulting crude organic extracts were filtered, vacuum-dried, and transferred into a flash column packed with 30 g of C18 material for desalting. After washing with 600 mL milliQ water^®^, it was eluted with 1200 mL of MeOH and dried under a vacuum to produce the solvent dipping extracts (SD). For the solid-phase (C-18) adsorption (SA), the algal thalli were covered with C18 material (Sepra C18-E, 50 µm, 65 A, Phenomenex^®^, Torrance, CA, USA) and agitated (alga to C18 material ratio, 5:1) for 2 min, and left at room temperature for 10 min [33]. The C18 material was then washed off the thalli and packed into a glass column. It was sequentially washed with 3-column volumes of seawater, milliQ water^®^, and 6-column volumes of MeOH. The MeOH phase was dried under a vacuum to yield solid-phase adsorption extracts (SA).

The algal material, which remained after both the SD and SA surface extractions, as well as the whole algal thalli (without surface extraction), were freeze-dried and pulverized using the speed rotor mill Pulverisette 14 (1.0 mm sieve ring, Fritsch GmbH, Idar-Oberstein, Germany). For each replicate (5 replicates per *Fucus* sp.), 4 g of algal material was extracted using the Accelerated Solvent Extractor system ASE 350^TM^ (Dionex, Thermo Fisher Scientific, Sunnyvale, CA, USA). Extractions were performed by using MeOH and DCM as described previously by Heavisides et al. [46]. A three-step extraction was performed with a water pre-rinse 10 min static (3 cycles), MeOH 5 min static (1 cycle), and DCM 5 min static (1 cycle). Samples were packed with acid-washed sand (Grüssing GmbH, Filsum, Germany) into 100 mL stainless steel cells and held at a temperature of 40 °C, with a purge time of 250 sec and rinse volume of 30% cell volume. The MeOH and DCM extracts were pooled, vacuum-dried, and named as surface-free extracts after C18 adsorption (SFA), surface-free extracts after solvent dipping (SFD), and whole algae extracts (W) without prior extraction of the surfaces.

### 4.3. UPLC-QToF -MS/MS Metabolomics

Aliquots (1 mg/mL in MeOH) of all the extracts, W, SFA, SFD, SD, and SA, including the solvent controls, were injected (0.3 µL) into an Acquity UPLC I-Class system connected to a Xevo G2-XS QToF-MS (Waters^®^, Milford, MA, USA). A binary mobile phase (MP) composed of ULC/MS grade solvents (VWR^®^, Radnor, PA, USA), water (A), and acetonitrile (B), both spiked with 0.1% formic acid (*v*/*v*). Metabolite separation was achieved on a C18 column (Acquity UPLC HSS T3, 1.8 μm, 2.1 × 100 mm, Waters^®^) at 40 °C with the MP infused at a flow rate of 0.6 mL/min and a gradient as follows: 1% B for 0.7 min, increased to 30% B from 0.7 to 1 min, increased further to 99% B from 1 to 13.50 min, followed by an isocratic step of 99% B for 5 min, back to the initial conditions within 0.5 min, and a column reconditioning step for 2 min, for a total run time of 21 min. MS was performed in fast DDA acquisition mode, with an ESI source over a mass range of *m*/*z* 50–1200 Da in both positive and negative polarities, with a capillary voltage of 800 V, cone gas flow of 50 L/h, desolvation gas flow of 1000 L/h, source temperature of 150 °C, and desolvation temperature of 500 °C, with the sampling cone and source offset at 40 and 80, respectively. The MS/MS experiment was achieved by using a collision energy ramp with the following settings: LM CE ramp start = 20, LM CE ramp end = 40, HM CE ramp start = 60, HM CE ramp end = 80, and a scan rate of 0.1 sec in centroid data format. All measurements were performed in quadruplicate and mass-corrected with LockSpray (reference masses: 120.0813 and 556.2771 Da MSMS for Leucine enkephalin). Data from the positive polarity mode were more diverse and so were used for statistics. All data were analyzed using MassLynx^®^ software (v4.2).

### 4.4. Molecular Networking

ProteoWizard msconvert (version 3.0.20033; Vanderbilt University, Nashville, TN, USA) [89] was employed to convert all .raw data files from the UPLC-MS/MS to mzXML format, which were then processed using MZmine 2.33 [90]. The MZmine pre-processing of all the raw MS (+) data resulted in 6325 *m*/*z* features. After applying several filtering steps (incl. removal of solvent-derived features), the final data set comprised 366 features from all the extracts (Table S1). The metabolomic features, including retention time and *m*/*z* and peak areas, were exported as .csv (feature quantitative table), while the MS^2^ data were exported as .mgf files. The pre-processed data (.csv and .mgf files) were uploaded onto the GNPS platform [26] to generate molecular networks using the Feature-Based Molecular Networking (FBMN) workflow [91]. Here, identical MS/MS spectra are combined into ‘consensus’ spectra and displayed as nodes. The nodes are then linked with edges based on the similarity between the consensus spectra using spectral alignment algorithms. A molecular network was created with the precursor ion and fragment ion mass tolerances set at 0.05 Da, with the edges filtered to have a cosine score above 0.7. The spectra were also searched against the GNPS spectral libraries (score > 0.6) [92]. The spectral network was further subjected to in silico tools, network annotation propagation (NAP) [93], and Dereplicator+ [94] to enhance the results of the annotations. The outputs from molecular networking, NAP, and Dereplicator+ were merged through the MolNetEnhancer [95] workflow, which employs ClassyFire [96] chemical taxonomy for chemical class annotation. The integrated network was visualized and analyzed using Cytoscape version 3.7.2 [97]. Additionally, the peak ions were manually analyzed using MassLynx version 4.2 to predict the molecular formulae and searched against the databases NP Atlas (https://www.npatlas.org (accessed on 3 May 2023)) and Dictionary of Natural Product (http://dnp.chemnetbase.com (accessed on 3 May 2023)).

### 4.5. DNA Extraction

For the analysis of the epiphytic microbial community, the thallus surfaces of each *Fucus* sp. were sampled for amplicon sequencing of the conserved V3-V4 region of the bacterial 16S rRNA gene and the fungal internal transcribed spacer region. To avoid any change in the microbial community, the samples for amplicon sequencing were processed immediately after arrival at the home laboratory. The surfaces of the thalli were swabbed using a sterile cotton tip and kept at −80 °C in sterile Eppendorf tubes. All algal samples were made in triplicate, resulting in a total of 54 algal samples (18 samples per species). Ambient seawater (500 mL, SW) was filtered through cellulose nitrate filters (pore size 0.45 µm, Whatman) in 6 replicates. Biofilm samples (Bf) were taken from a stone laying in the *Fucus* meadow in 6 replicates. All samples were stored at −80 °C prior to DNA extraction. Genomic DNA was extracted using the DNeasy PowerSoil Kit (Qiagen, Hilden, Germany). DNA concentration was measured prior to sequencing using a Nanovue UV-Vis spectrophotometer (GE Healthcare, USA) and stored at −80 °C.

### 4.6. Amplicon Sequencing and Bioinformatic Processing

PCR, purification, library preparation, and Illumina sequencing were implemented at Novogene Europe Ltd. (Cambridge, UK), using the upgraded NovaSeq sequencing platform (2 × 250 bp). The hypervariable regions V3-V4 of the bacteria 16S rRNA gene were amplified with primers 341F (5′-CCTAYGGGRBGCASCAG-3′) and 806R (5′- GGACTACNNGGGTATCTAAT -3′). The ITS2 region of the fungi were amplified with primers ITS3-2024F (5′-GCATCGATGAAGAACGCAGC-3′) and ITS4-2409R (5′-TCCTCCGCTTATTGATATGC-3′). Based on the unique barcodes, the paired-end reads were assigned to samples before cutting off the barcodes and primer sequences. The paired-end reads were merged using FLASH (Version 1.2.11) [98] to obtain Raw Tags, which were filtered to obtain clean tags with fastp (Version 0.20.0). The cleaned sequences were further processed using the DADA2 package (version 1.26.0) [99]. After filtering and trimming steps, the forward and reverse reads were merged and the chimeras were removed using the removeBimera function. For taxonomy assignment, the IdTaxa function in the DECIPHER package (version 2.26.0) [100] was used with SILVA_SSU_r138_2019 as the reference database for the bacterial sequences, and UNITE_v2021_May2021 as the reference database for the ITS sequences. From the bacterial dataset, chloroplast sequences were removed (no mitochondrial sequences detected); from the ITS dataset, sequences assigned to Anthophyta and Phaeophyceae were removed. A subsequent analysis was performed using the phyloseq package (v.1.42.0) [101]. The removal of samples below 15,000 reads and rarefying to 15,112 reads led to the exclusion of two samples (one each from *F. serratus* and *F. distichus* subsp. *evanescens*) for further analysis of the 16S data. Due to low DNA concentrations, the ITS fragment was not sequenced for 4 samples from the BF, and data reduction steps eliminated a further two samples (one each from the SW and *F. distichus* subsp. *evanescens*), resulting in a total of 60 samples. The ITS data were rarefied to 84,856 reads. For both datasets, alpha diversity was calculated for Observed ASVs and the Shannon index, and statistically assessed using the Wilcoxon rank test. After determining the Bray–Curtis dissimilarity index as the best method with which to assess beta diversity, it was visualized using NMDS plots. Effects on community dissimilarity were analyzed using a permutational analysis of variance (PERMANOVA; Anderson, SC, USA, 2001) with the adonis2 function (vegan R package). A Linear discriminant analysis effect size (LEfSe) was performed using microeco package (v.0.13.0) [102], and the rarefaction curves were calculated using the microdev extension mecodev (v.2.0). Raw amplicon sequences were deposited in the Sequence Read Archive of the NCBI, with accession number PRJNA635604.

### 4.7. Statistical Analysis

A multivariate statistical analysis of the metabolomics data (LC-(+)-ESI-MS) was achieved on the MetaboAnalyst platform (version 5.0) [103]. Sample data were normalized using the median and scaled using Auto scaling (mean centered and divided by the standard deviation of each variable) prior to statistical analysis and visualization. The PLS-DA model generated variable importance in projection (VIP) scores, which were used for biomarker prediction [83].

## 5. Conclusions

In conclusion, we comparatively analyzed the surface metabolome and the epibiome of three co-occurring *Fucus* spp., *F. distichus* subsp. *evanescens*, *F. vesiculosus*, and *F. serratus*, to gain insights into the potential factors underlying the observed differential fouling levels. The surface metabolites were clearly segregated from the tissue metabolites, with many common metabolites among the three spp. FBMN and multivariate statistical analyses revealed many species-specific and discriminant surface metabolites, such as MGTAs, DGTAs, and carboxylic acid derivatives, which contribute to the chemical differences of the algal surfaces. This study also provides evidence that the *Fucus* spp. differentially harbor diverse epiphytic prokaryotic and eukaryotic communities influencing the surface biofilm. The species-specific and discriminant metabolites, such as ulvaline, MGTA 20:5, MGTA 20:4, 4-pyridoxic acid, taxa (*Yoonia-Loktanella*, *Alternaria*, and Mucoromycota), and other factors, such as the thallus morphology of *F. distichus* subsp. *evanescens*, may directly and/or indirectly contribute to the least fouled surface observed. Our results show the ecological importance of some epibionts and surface metabolites for the *Fucus* spp., and also have implications for the development of antifouling strategies and the conservation of coastal habitats.

## Data Availability

The raw reads from the sequencing of the 16S rDNA V3-V4 region amplicons and ITS2 amplicons have been deposited into the Sequence Read Archive (SRA) of the National Center for Biotechnology Information (NCBI) database (BioProject: PRJNA992394). The MS data used for the molecular networking analysis were deposited into the MassIVE Public GNPS database under the accession number MSV000092347. The molecular networking job can be publicly accessed at https://gnps.ucsd.edu/ProteoSAFe/status.jsp?task=b56a28db5d8f4199b495e585c454a529 (accessed on 6 October 2023).

## Supplementary Materials

The following supporting information can be downloaded at: https://www.mdpi.com/article/10.3390/md21110595/s1, Figures S1–S5: UPLC-MS base peak chromatograms of organic extracts; Figure S6: MN generated from UPLC-(-)-ESI-MS/MS data; Figure S7: Variation in the most significant discriminatory metabolite markers; Figures S8–S10: MS/MS spectra of annotated VIPs; Figure S11: PCA scores plot; Figure S12: MN of surface-free and whole extracts; Figure S13: Rarefaction curves of bacterial amplicon sequences; Figures S14 and S15: Alpha diversity of bacterial epiphytic community; Figure S16: Beta diversity analysis of bacterial amplicon data; Figure S17: Bacterial orders associated with the surfaces of the *Fucus* spp.; Figure S18: Rarefaction curves of eukaryotic ITS-region amplicon sequences; Figures S19 and S20: Alpha diversity of eukaryotic epiphytic community; Figures S21 and S22: Alpha diversity of fungal epiphytic community; Figure S23: Fungal orders associated with the surfaces of the *Fucus* spp., and the stone biofilm (BF) and seawater (SW) reference samples; Table S1: Putative annotations of metabolites; Table S2: Relative abundances of bacterial genera (>1%) associated with the surfaces of the *Fucus* spp., seawater, and stone biofilm; Table S3: Bacterial beta diversity statistics; Table S4: Relative abundances of eukaryote genera (>1%) associated with the surfaces of the *Fucus* spp., seawater, and stone biofilm; Table S5: ITS beta diversity statistics; Table S6: Relative abundances of fungal genera (>1%) associated with the surfaces of the *Fucus* sp., seawater, and stone biofilm.
