# Supplementary material for: Metabolomics and Microbiomics Insights into Differential Surface Fouling of Three Macroalgal Species of Fucus (Fucales, Phaeophyceae) That Co-Exist in the German Baltic Sea
Source: Mar Drugs. 2023 Nov 16;21(11):595. doi: 10.3390/md21110595 (PMC10672516; doi:10.3390/md21110595)
Supplement: Supplementary file 1 [file marinedrugs-21-00595-s001.zip › Tasdemir_Fucus_MDrugs_SI.pdf]

Supplementary Information

Metabolomics and Microbiomics Insights into Differential Surface Fouling of Three Macroalgal Species of *Fucus* (Fucales, Phaeophyceae) that Co-exist in the German Baltic Sea

Ernest Oppong-Danquah [1], Martina Blümel [1] and Deniz Tasdemir [1,2,*]

[1] GEOMAR Centre for Marine Biotechnology (GEOMAR-Biotech), Research Unit Marine Natural Products Chemistry, GEOMAR Helmholtz Centre for Ocean Research Kiel, Wischhofstrasse 1-3, 24148 Kiel, Germany; eoppong-danquah@geomar.de (E.O.-D.); mbluemel@geomar.de (M.B.)
[2] Faculty of Mathematics and Natural Science, Kiel University, Christian-Albrechts-Platz 4, 24118 Kiel, Germany
* Correspondence: dtasdemir@geomar.de; Tel.: +49-431-6004430

List of Figures — Page No.

Figure S1: UPLC-MS(+) base peak chromatograms of Surface Adsorption (SA) extracts of A) FE, B) FS and C) FV 3
Figure S2: UPLC-MS(+) base peak chromatograms of Solvent Dipping (SD) extracts of A) FE, B) FS and C) FV 4
Figure S3: UPLC-MS(+) base peak chromatograms of Surface-free after adsorption (SFA) extracts of A) FE, B) FS and C) FV 5
Figure S4: UPLC-MS(+) base peak chromatograms of Surface-free after dipping (SFD) extracts of A) FE, B) FS and C) FV 6
Figure S5. UPLC-MS(+) base peak chromatograms of Whole (W) extracts of A) FE, B) FS and C) FV 7
Figure S6. MN generated from UPLC-(-)-ESI-MS/MS data of all extracts of *Fucus* spp. in the negative ion mode 8
Figure S7. Variation of the most significant discriminatory metabolite markers (VIP >1.8) on the surfaces of *Fucus* spp. 9
Figure S8. MS/MS spectra of $[M + H]^+$ ion of ulvaline at *m/z* 236.1 displaying the typical product ions 10
Figure S9. MS/MS spectra of $[M + H]^+$ ion of MGTA 20:4 at *m/z* 522.3 displaying the typical product ions 11
Figure S10. MS/MS spectra of $[M + H]^+$ ion of MGTA 18:1 at *m/z* 500.4 displaying the typical product ions 12
Figure S11. PCA scores plot generated from UPLC-(+)-ESI-MS data of all extracts 13
Figure S12. MN generated from UPLC-(+)-ESI-MS/MS data of all surface-free and whole extracts 14
Figure S13. Rarefaction curves of bacterial V3/V4 region amplicon sequences from all 66 samples 15
Figure S14. Alpha diversity (ASV-Observed vs. Shannon) of bacterial epiphytic community with regard to sample source 16

Figure S15. Alpha diversity (ASV-Observed vs. Shannon) of bacterial epiphytic community with regard to individual. 17
Figure S16. Beta diversity analysis of bacterial amplicon data based on Bray-Curtis distance calculation on *Fucus* spp. 18
Figure S17. Bacterial orders associated with surfaces of *Fucus* spp. 19
Figure S18. Rarefaction curves of eukaryotic ITS region amplicon sequences from all 60 samples. 20
Figure S19. Alpha diversity (ASV-Observed vs. Shannon) of eukaryotic epiphytic community based on ITS fragment sequences with regard to sample source. 21
Figure S20. Alpha diversity (ASV-Observed vs. Shannon) of eukaryotic epiphytic community based on ITS fragment sequences with regard to individual 22
Figure S21. Alpha diversity (ASV-Observed vs. Shannon) of fungal epiphytic community based on ITS fragment sequences with regard to sample source. 23
Figure S22. Alpha diversity (ASV-Observed vs. Shannon) of fungal epiphytic community based on ITS fragment sequences with regard to individual. 24
Figure S23. Fungal orders associated to surfaces of *Fucus* spp., stone biofilm (BF) and seawater (SW) reference samples 25

List of Tables — Page No.

Table S2. Relative abundances of bacterial genera (> 1%) associated to surfaces *Fucus* spp., Seawater and stone biofilm 26
Table S3. Bacterial beta diversity statistics based on Bray-Curtis dissimilarity 27
Table S4. Relative abundances of eukaryote genera (> 1%) associated to surfaces of *Fucus* spp., seawater and stone biofilm 28
Table S5. ITS beta diversity statistics based on Bray-Curtis dissimilarity 29
Table S6. Relative abundances of fungal genera (>1%) associated to surfaces of *Fucus* sp., seawater and stone biofilm 30

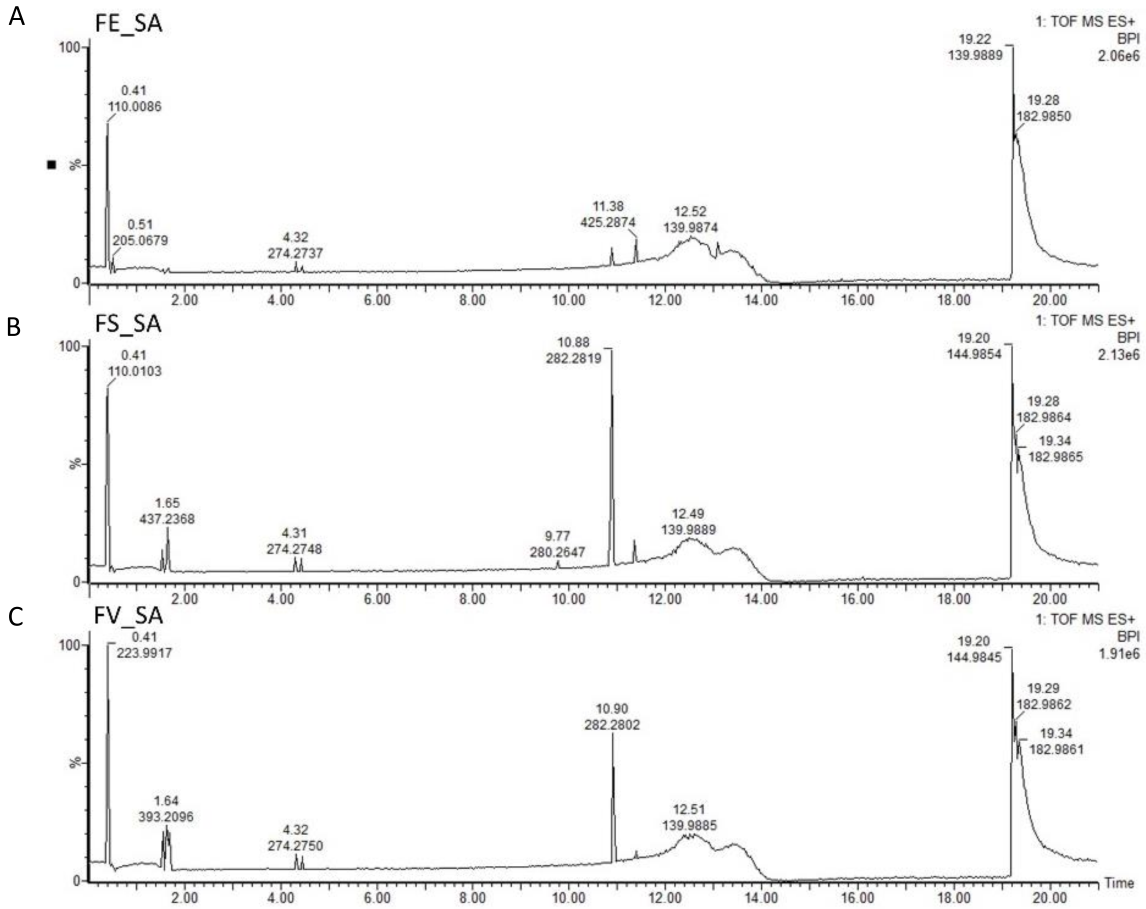

Figure S1. UPLC-MS(+) base peak chromatograms of Surface Adsorption (SA) extracts of A) FE, B) FS and C) FV

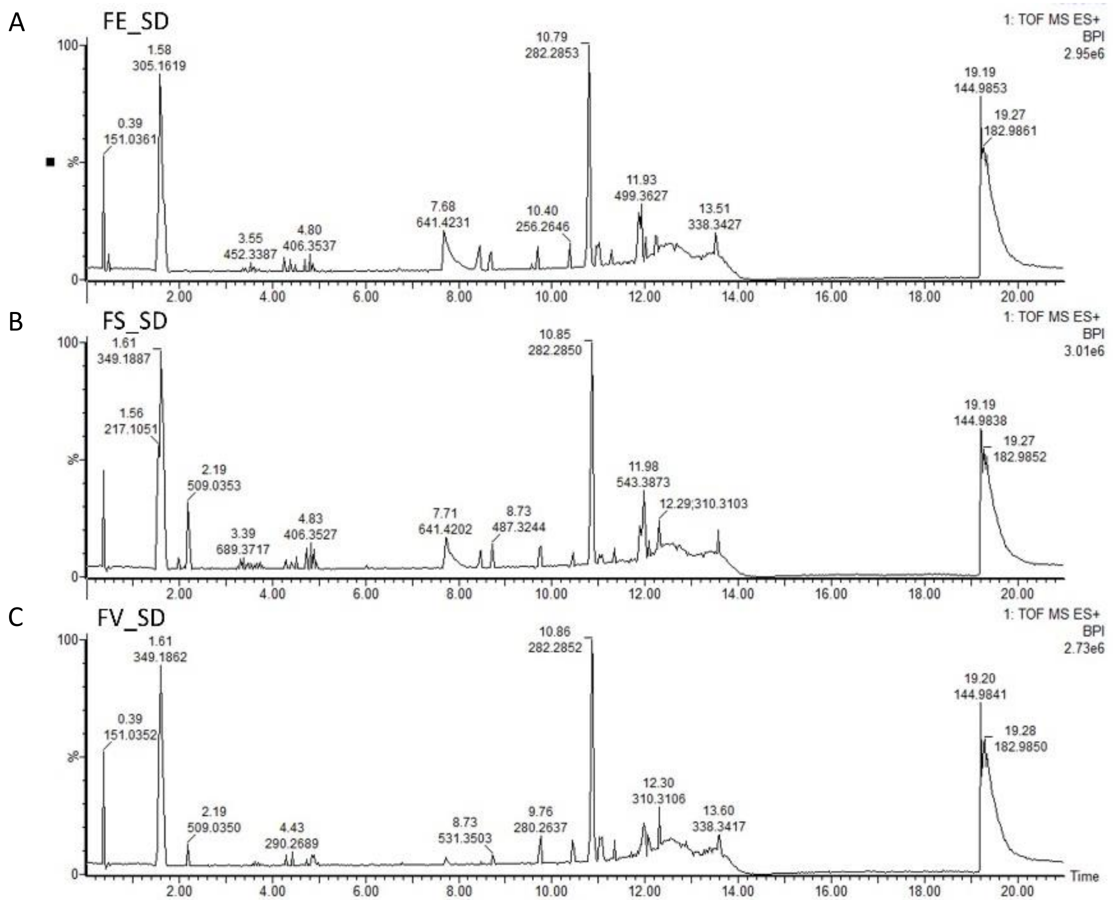

Figure S2. UPLC-MS(+) base peak chromatograms of Solvent Dipping (SD) extracts of A) FE, B) FS and C) FV

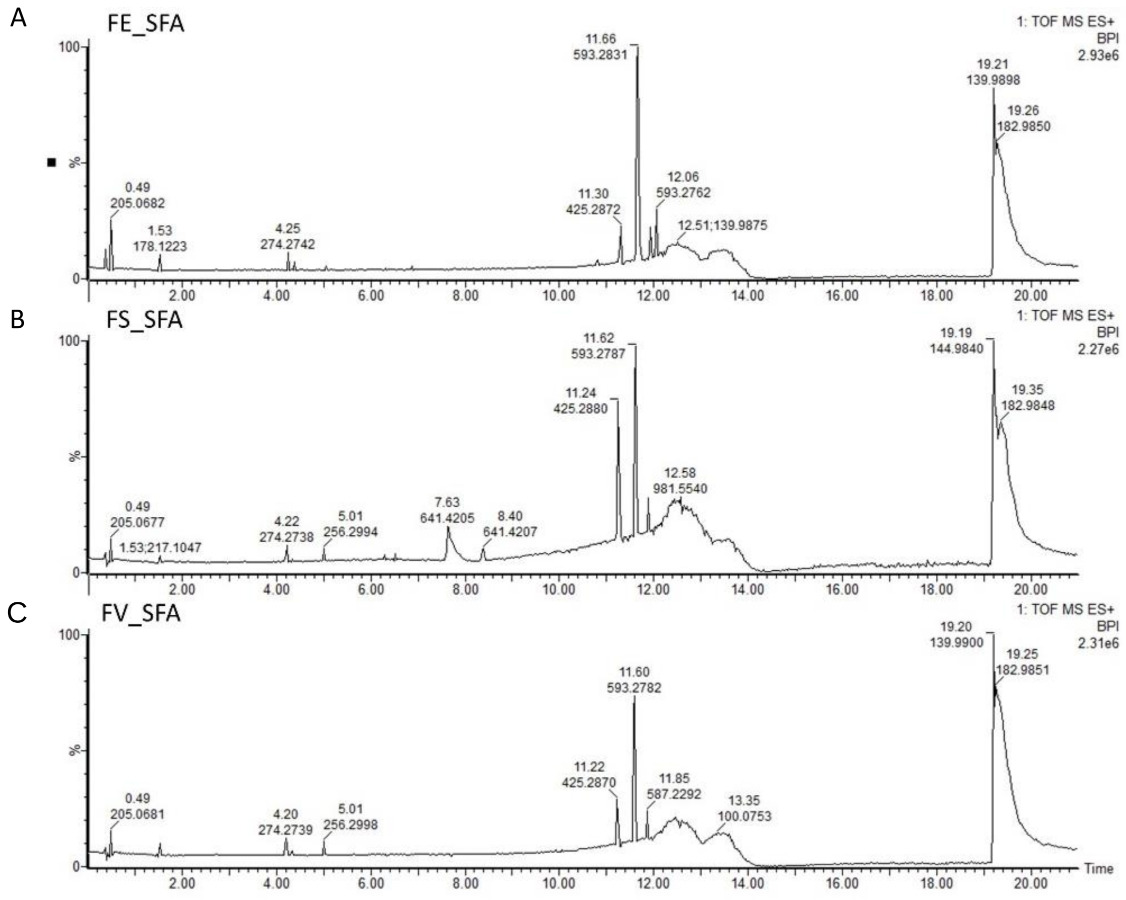

Figure S3. UPLC-MS(+) base peak chromatograms of Surface-free after adsorption (SFA) extracts of A) FE, B) FS and C) FV

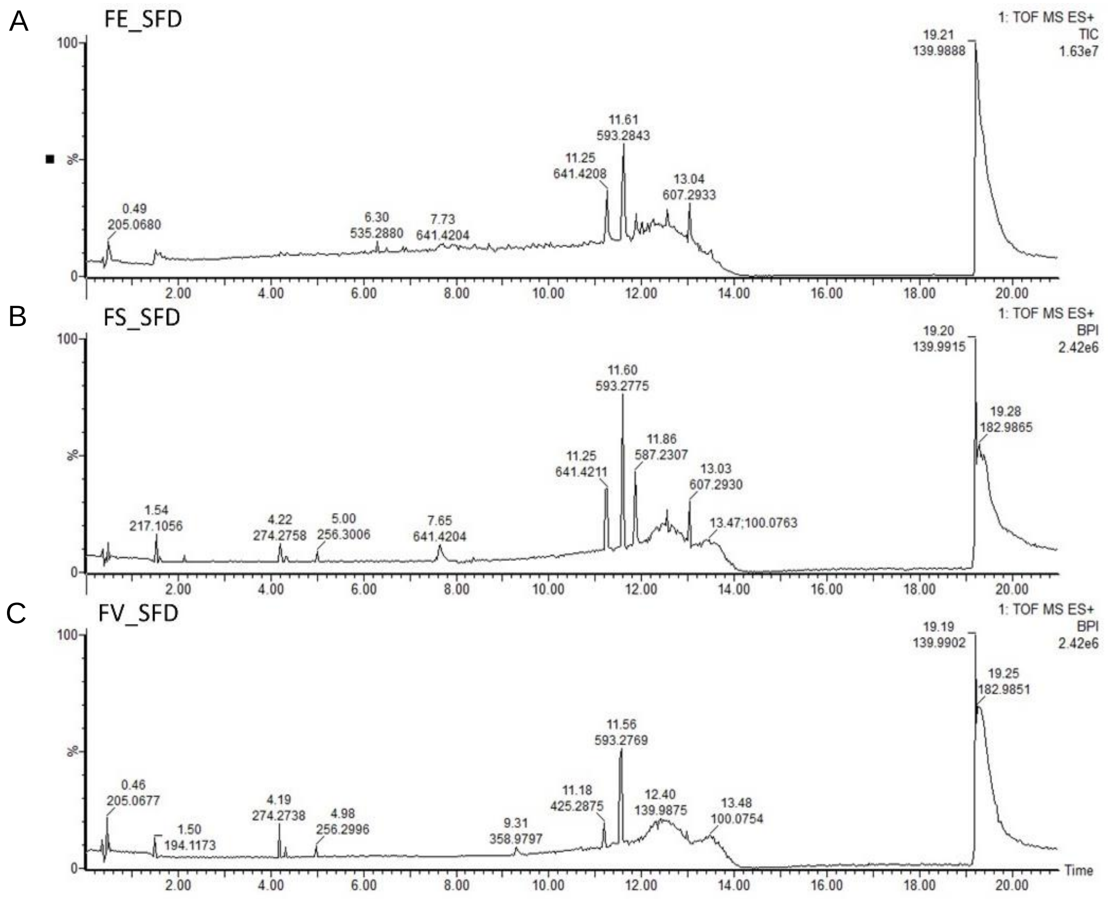

Figure S4. UPLC-MS(+) base peak chromatograms of Surface-free after dipping (SFD) extracts of A) FE, B) FS and C) FV

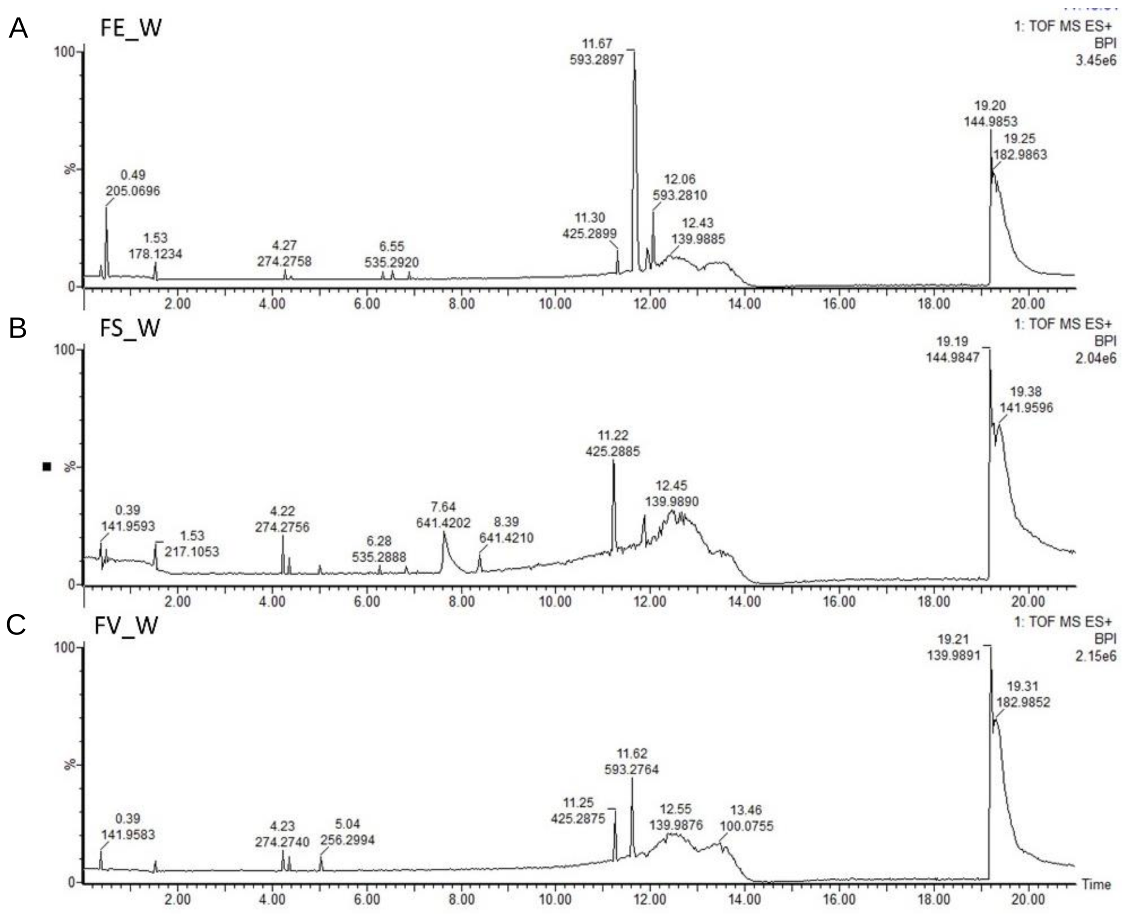

Figure S5. UPLC-MS(+) base peak chromatograms of Whole (W) extracts of A) FE, B) FS and C) FV

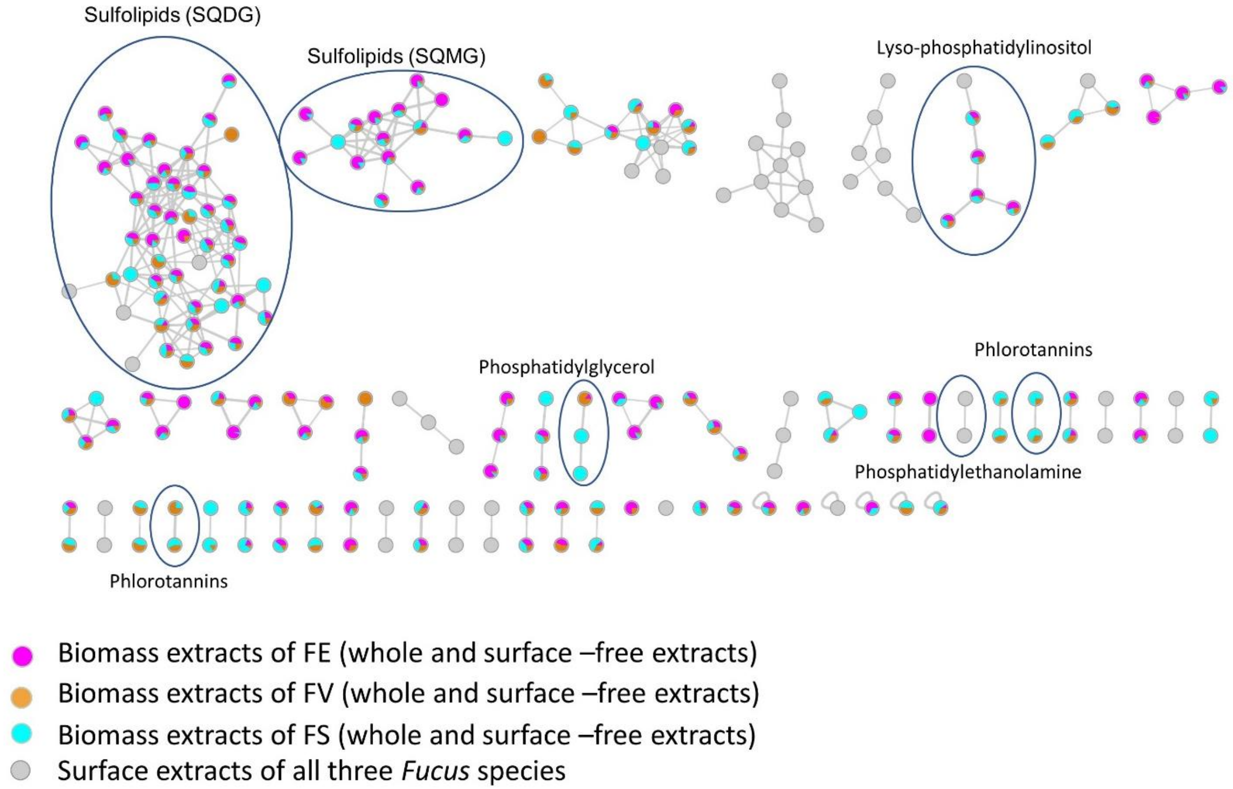

Figure S6. MN generated from UPLC-(-)-ESI-MS/MS data of all extracts of *Fucus* spp. in the negative ion mode. Node colour represents the source of the ion.

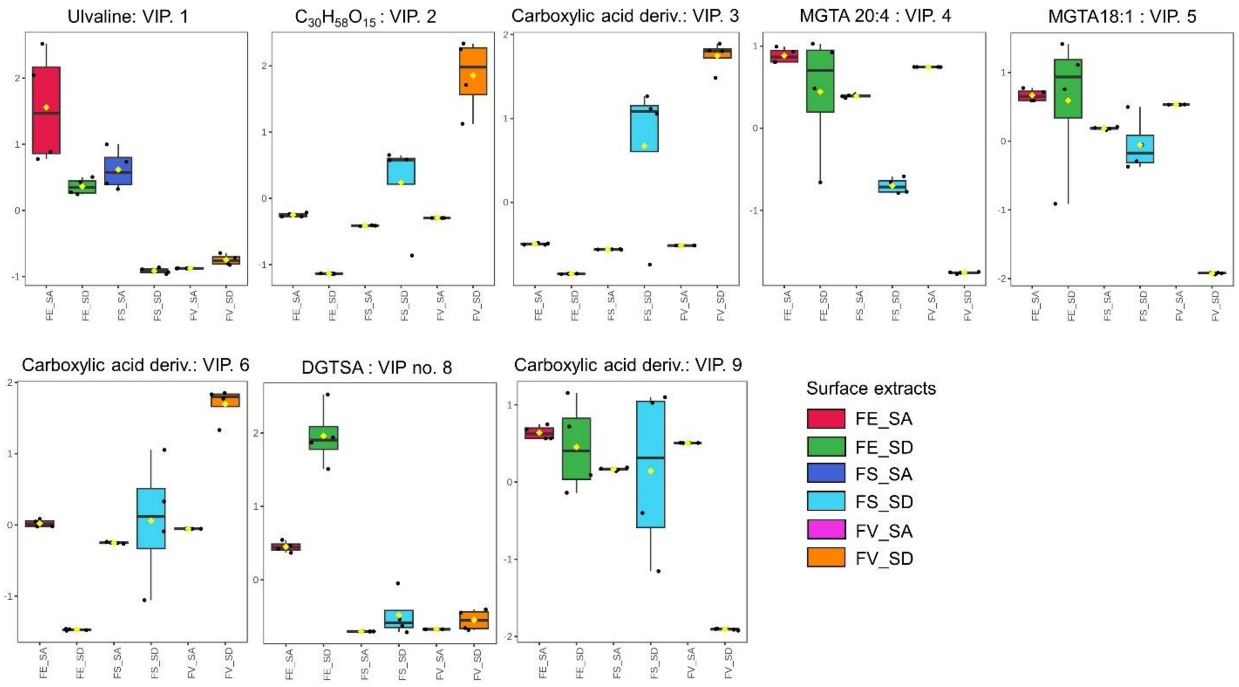

Figure S7. Variation of the most significant discriminatory metabolite markers (VIP >1.8) on the surfaces of the three *Fucus* spp. Normalized intensities are presented on the y-axis.

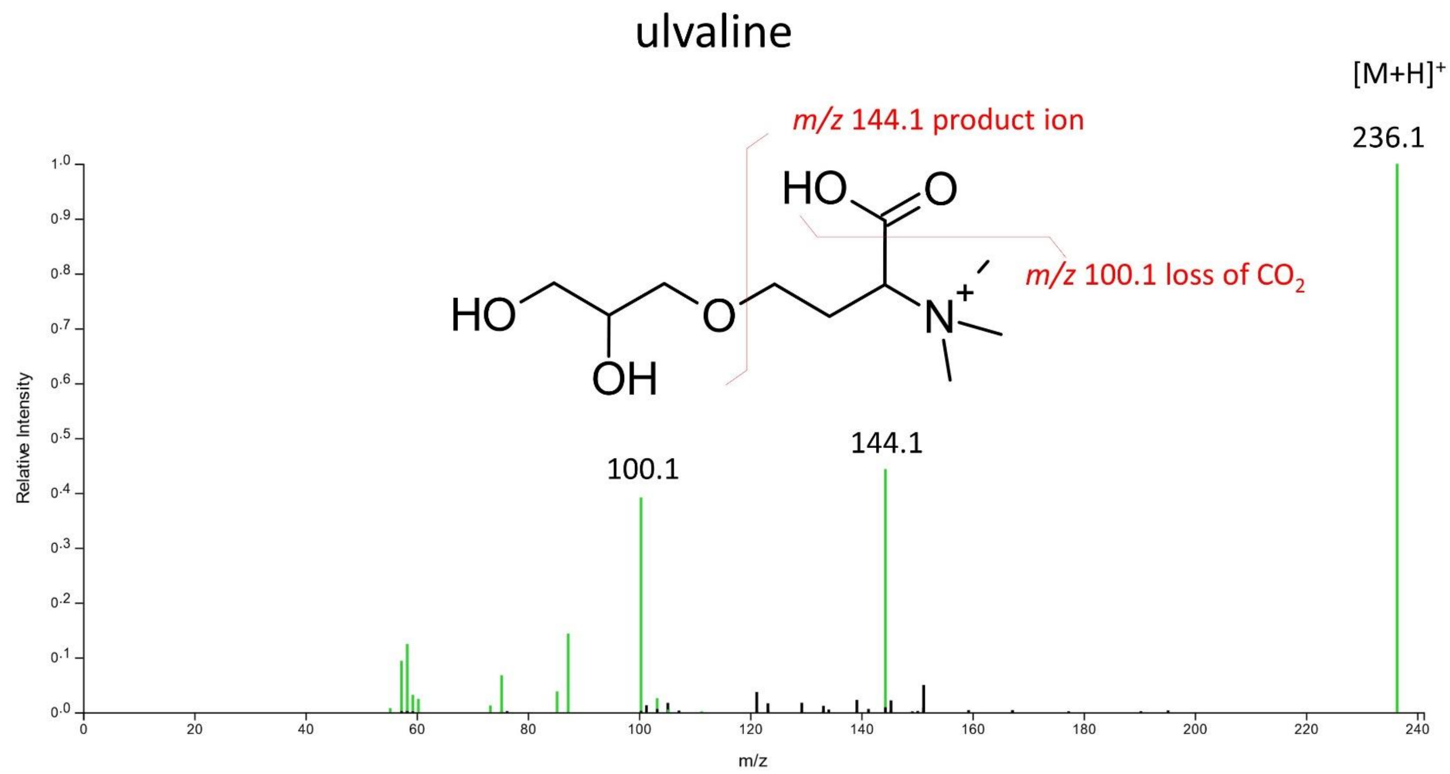

Figure S8. MS/MS spectra of [M + H]+ ion of ulvaline at *m*/*z* 236.1 displaying the typical product ions.

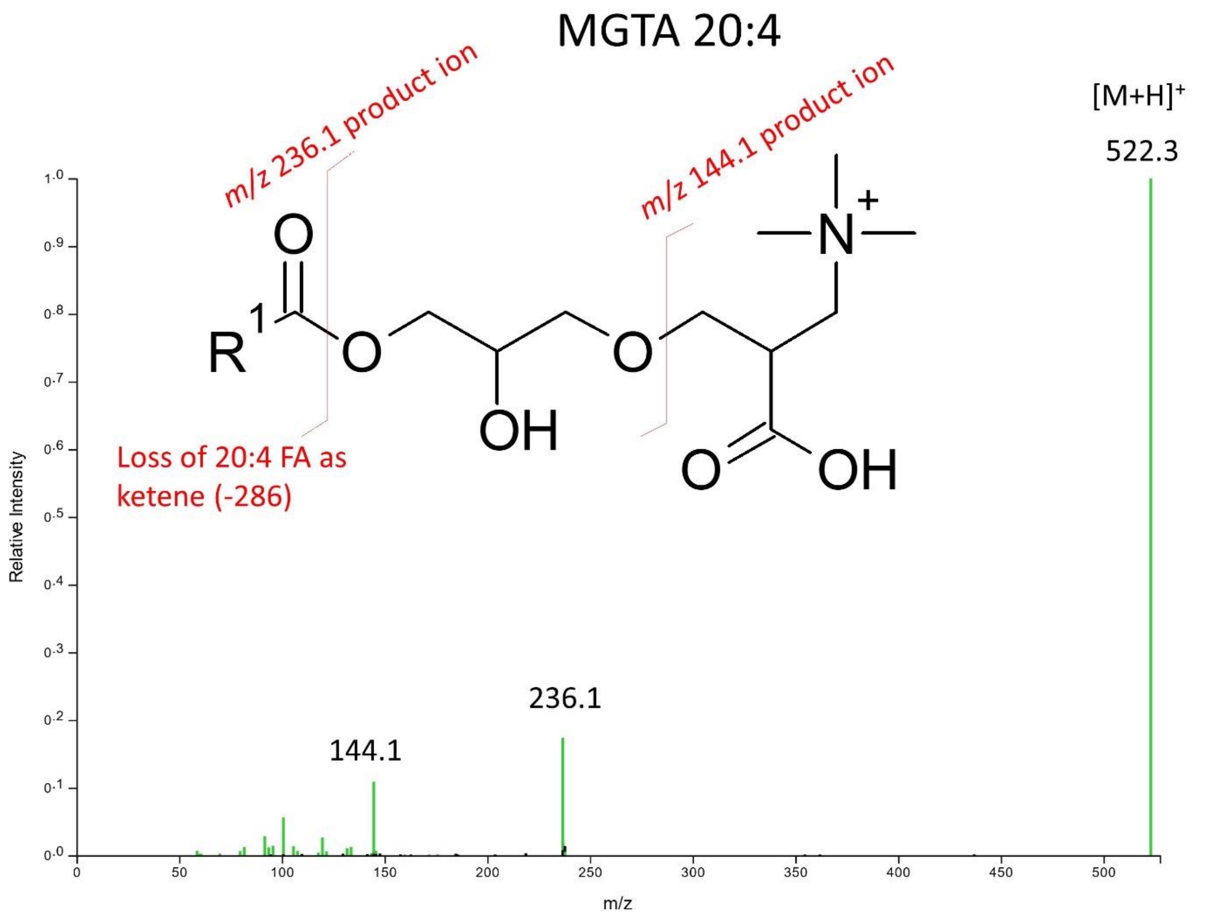

Figure S9. MS/MS spectra of $[M + H]^+$ ion of MGTA 20:4 at *m/z* 522.3 displaying the typical product ions.

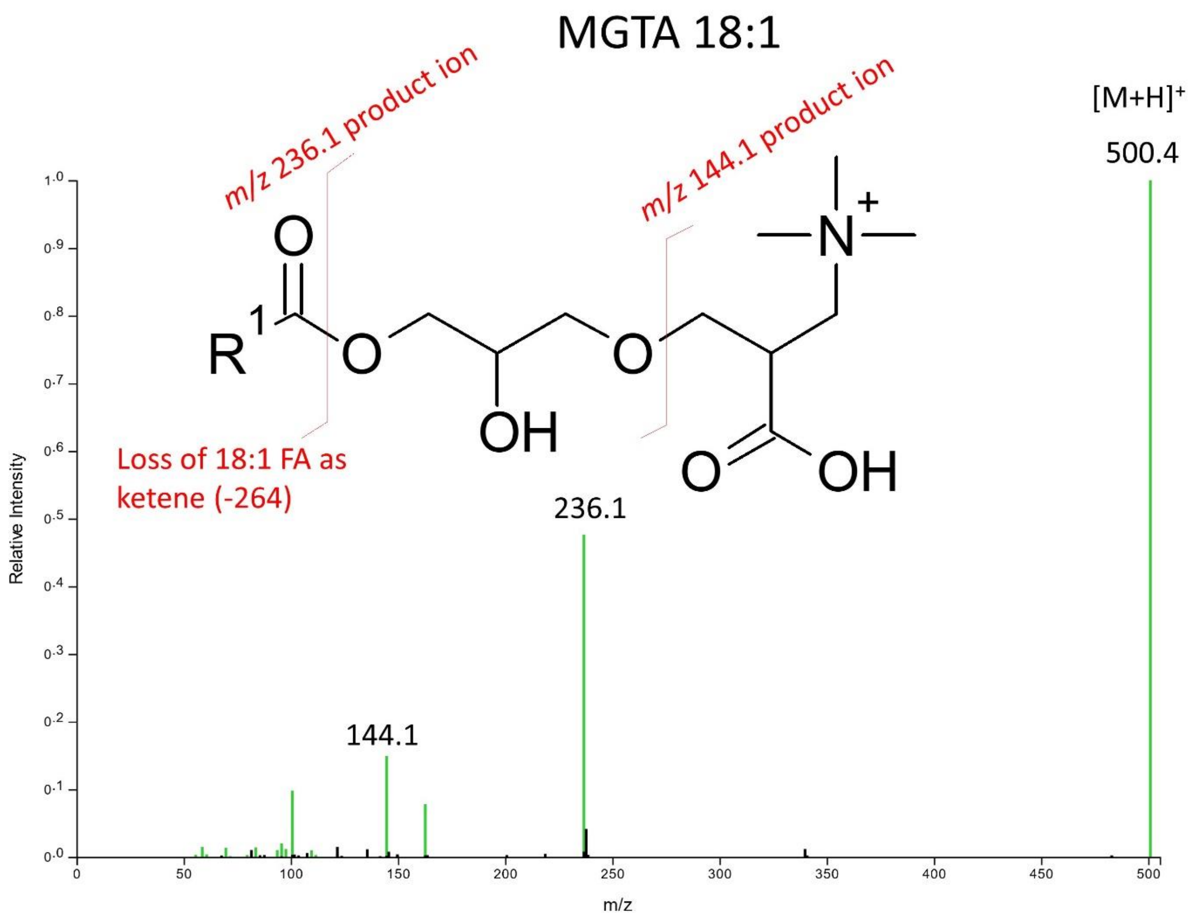

Figure S10. MS/MS spectra of $[M + H]^+$ ion of MGTA 18:1 at *m/z* 500.4 displaying the typical product ions.

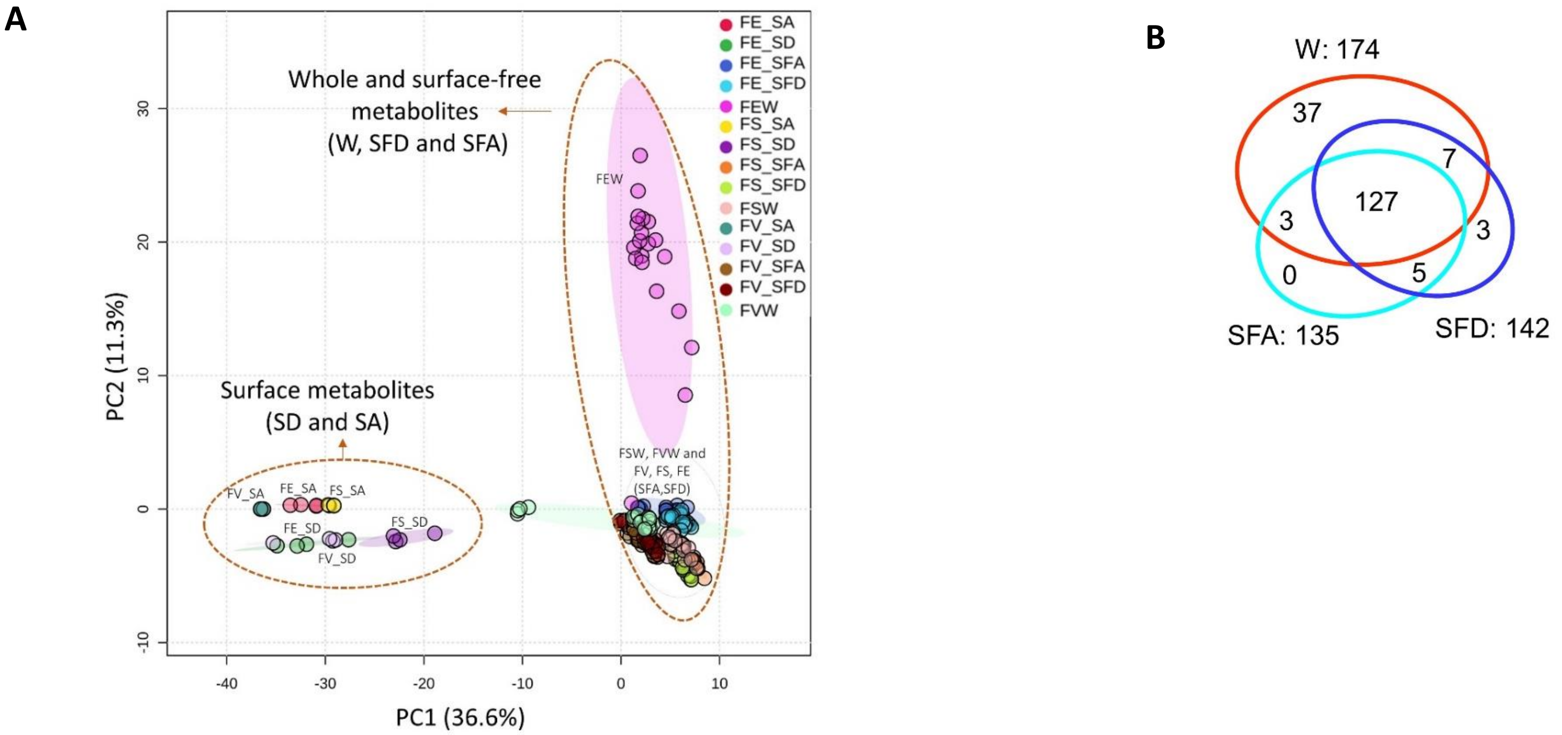

Figure S11. PCA scores plot generated from UPLC-(+)-ESI-MS data of all extracts showing a clear discrimination between surface metabolites from surface free and whole metabolome. Algal species: FV: *F. vesiculosus*, FS: *F. serratus*, FE: *F. distichus* subsp. *evanescens*, Extracts: SA: surface adsorption, SD: solvent dipping, SFA: surface-free after adsorption, SFD: surface-free after dipping, W: whole, untreated algae (B) Venn diagram showing distribution of nodes among the whole extracts (W), surface-free extract after adsorption (SFD) and dipping (SFD).

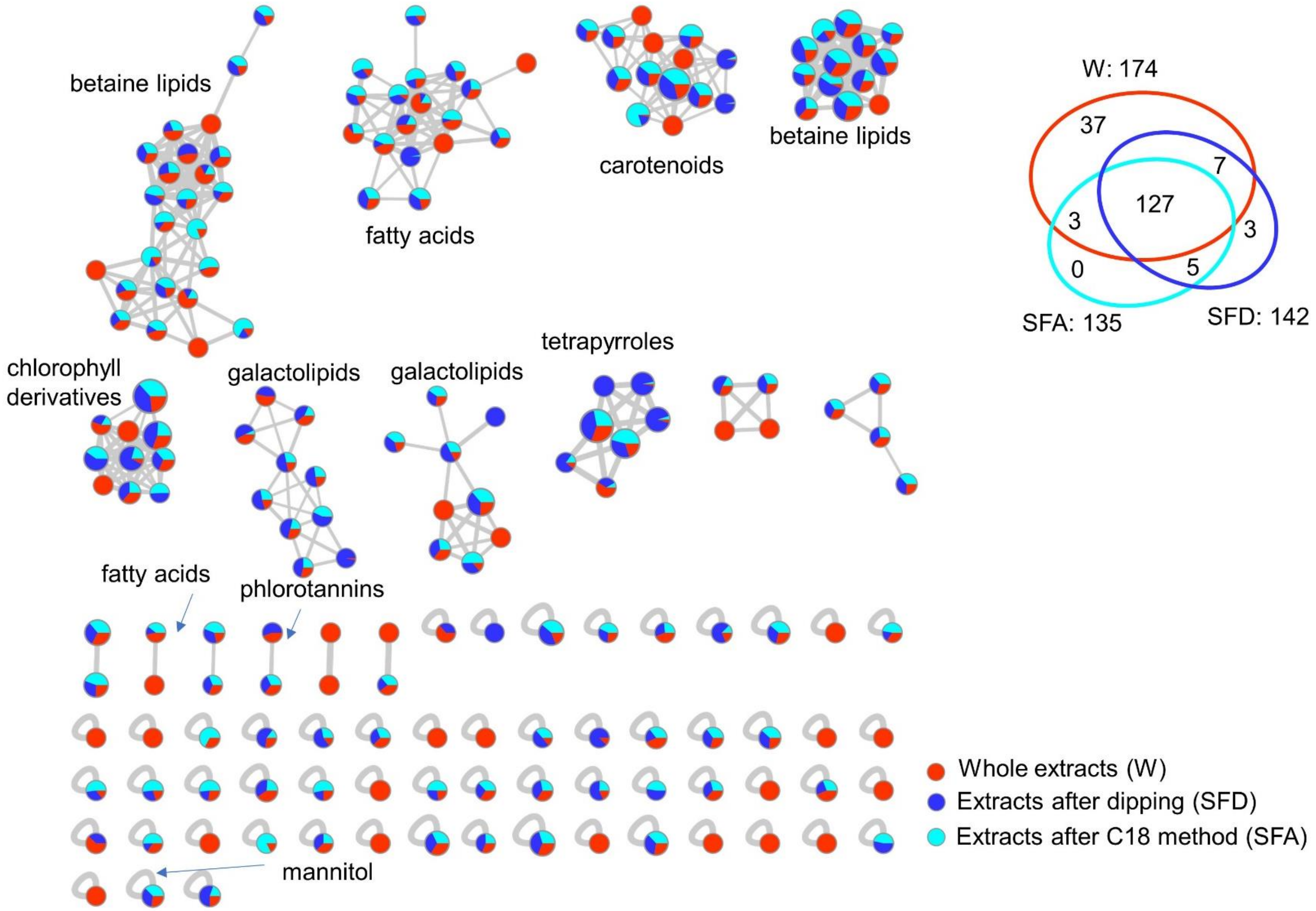

Figure S12. MN generated from UPLC-(+)-ESI-MS/MS data of all surface-free and whole extracts. Node sizes are modulated according to the sum of intensities of the ions in all extracts while the colors in the pie chart of each node represents the relative quantity of the ion from whole extracts (red), surface-free after dipping (blue) and surface-free after C18 adsorption (light blue)

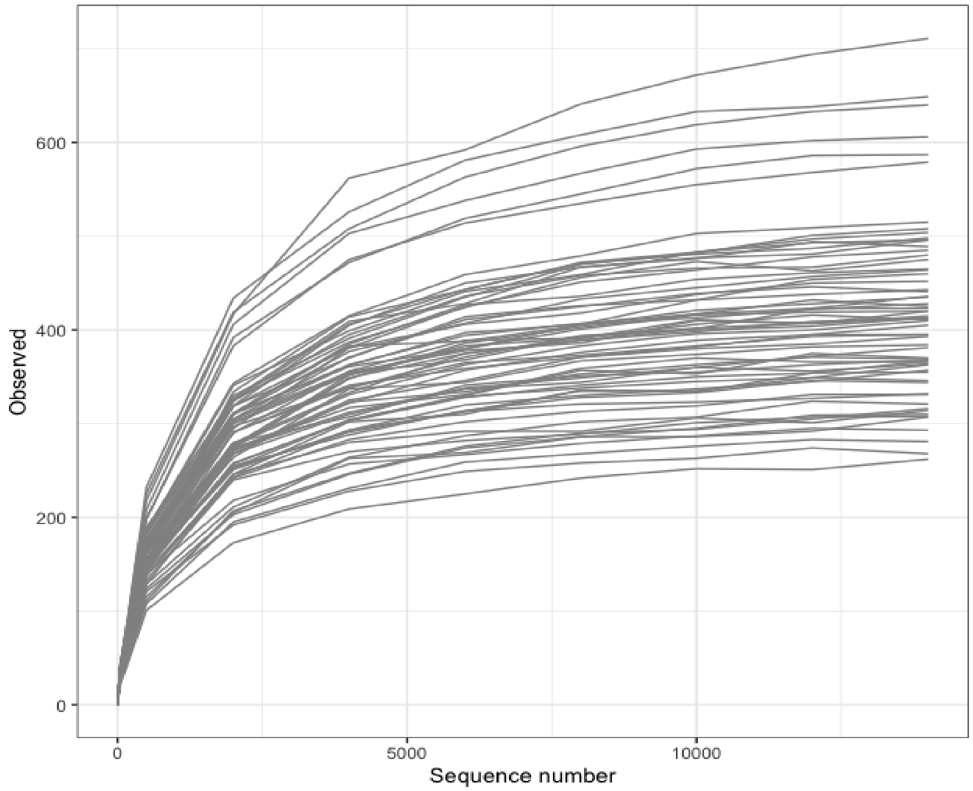

Figure S13. Rarefaction curves of bacterial V3/V4 region amplicon sequences from all 66 samples.

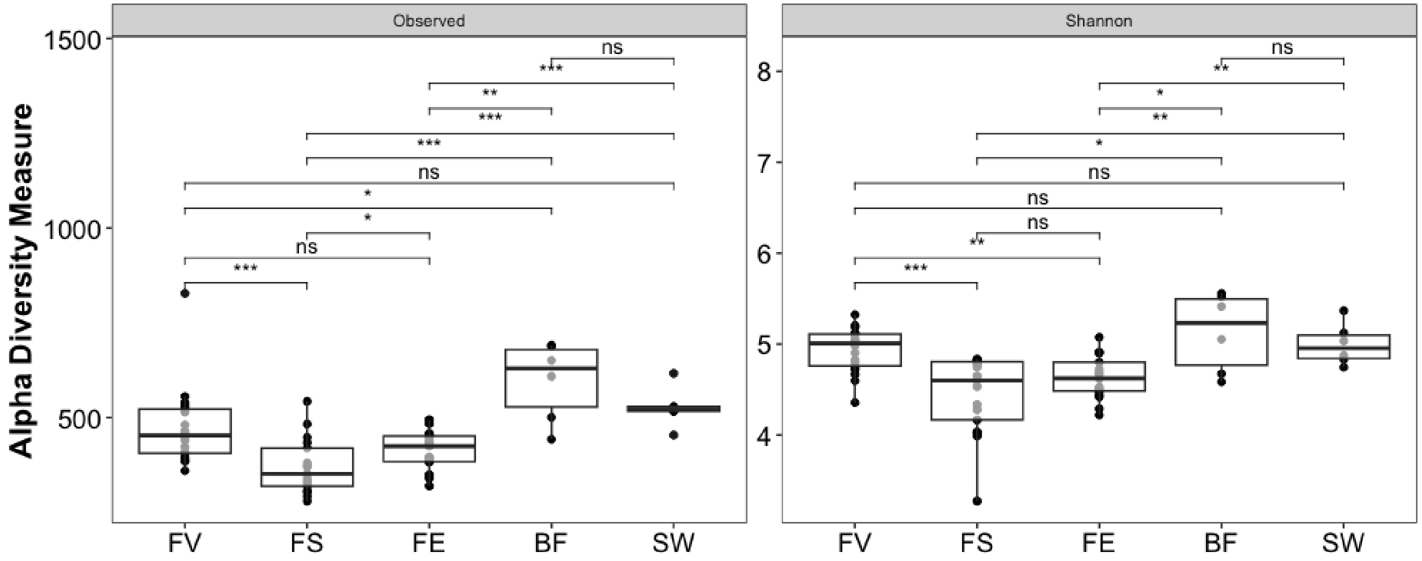

Figure S14. Alpha diversity (ASV-Observed vs. Shannon) of bacterial epiphytic community with regard to sample source. FV: *F. vesiculosus*, FS: *F. serratus*, FE: *F. distichus* subsp. *evanescens,* BF: biofilm on stone, SW: seawater. Significance levels: >0.0001: ***, >0.001: **, 0.01: *, >0.05: ns)

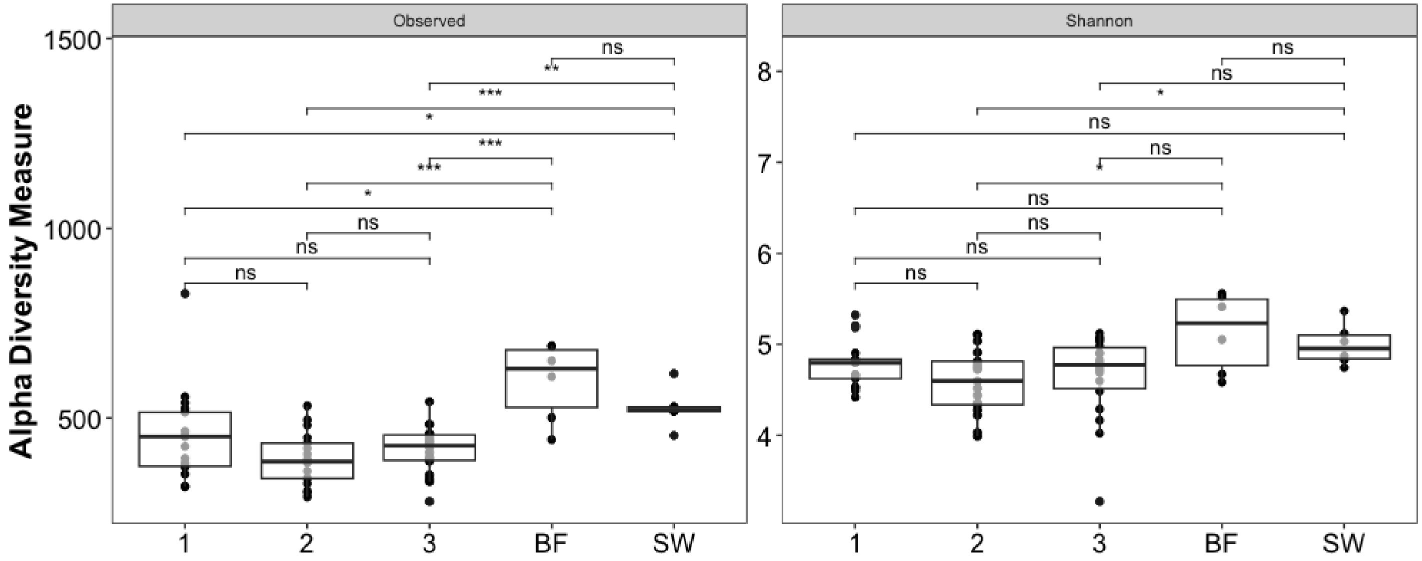

Figure S15. Alpha diversity (ASV-Observed vs. Shannon) of bacterial epiphytic community with regard to individual. 1: individual 1, 2: individual 2, 3: individual 3, BF: biofilm on stone, SW: seawater. Significance levels: >0.0001: ***, >0.001: **, 0.01: *, >0.05: ns)

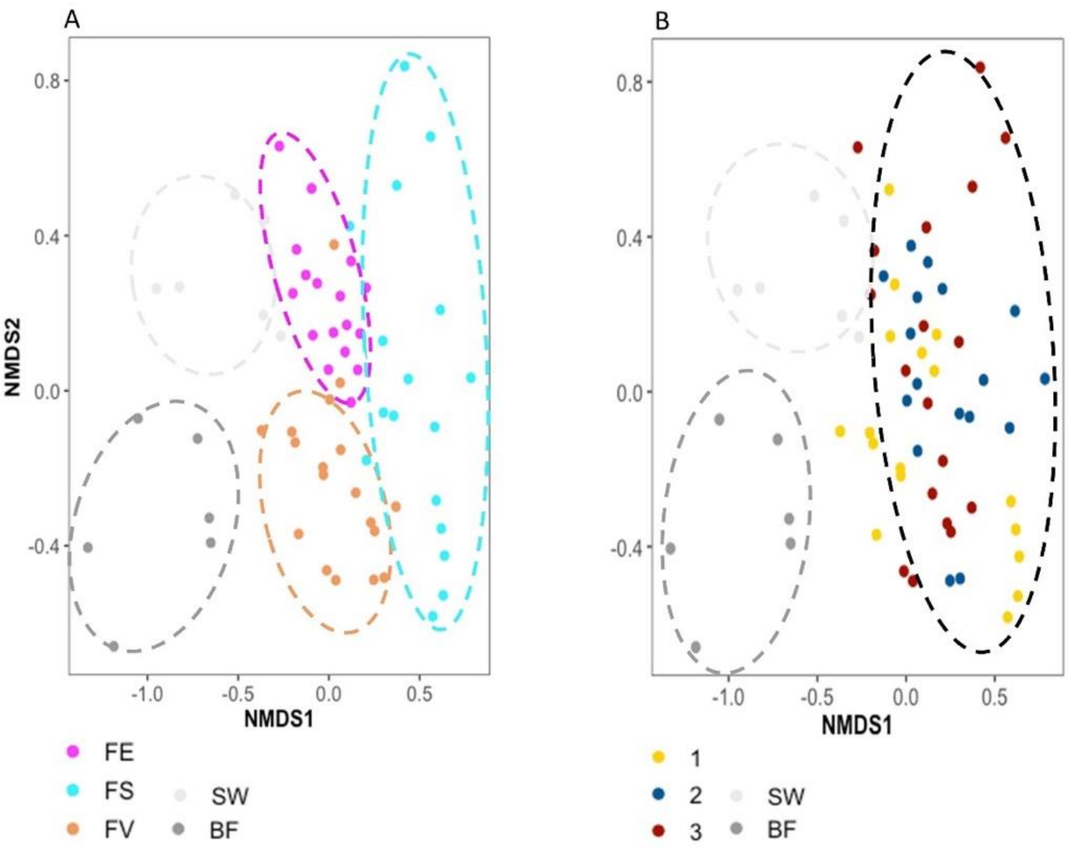

Figure S16. Beta diversity analysis of bacterial amplicon data based on Bray-Curtis distance calculation visualized by NMDS plots (A) according to sample origin (B) according to individual. FV: *Fucus vesiculosus*, FS: *F. serratus,* FE*: F. distichus* subsp. *evanescens,* BF: biofilm on stone, SW: seawater.

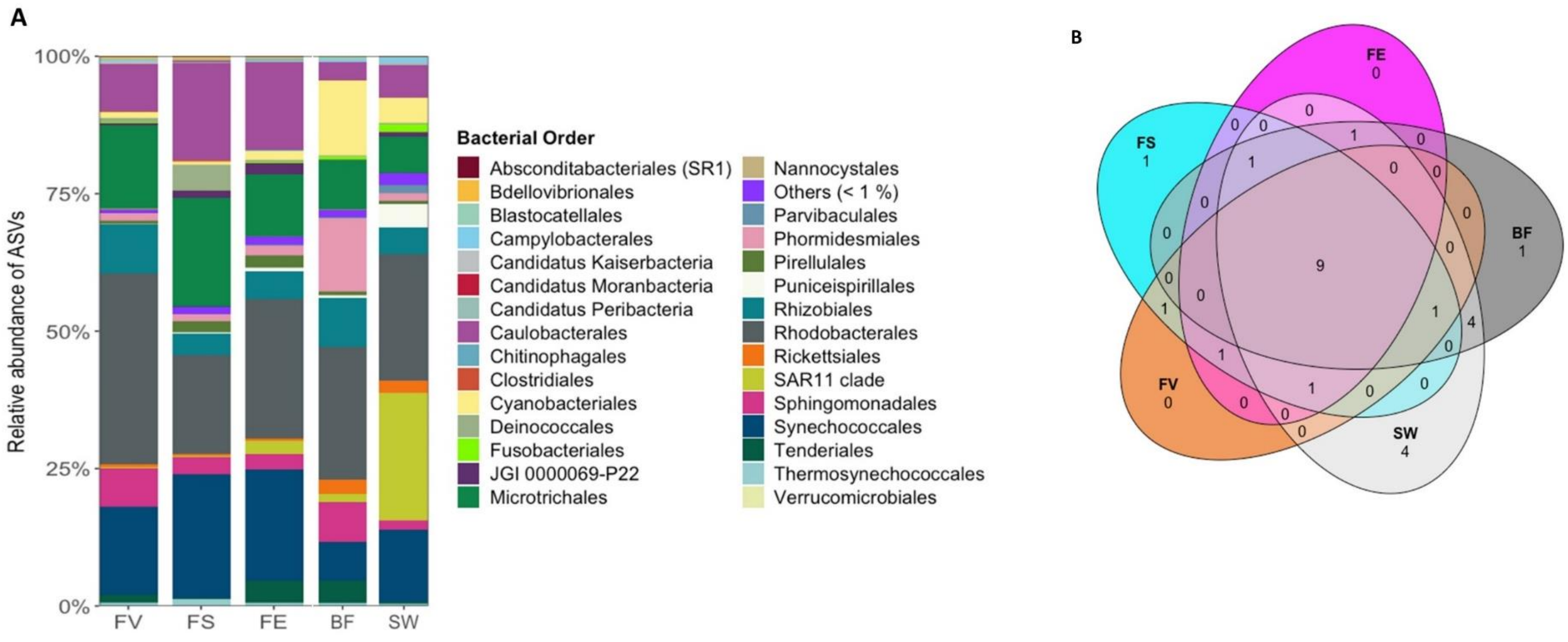

Figure S17. (A) Bacterial orders associated with surfaces of *Fucus* spp., and stone biofilm and seawater reference samples. Others (<1%) represents several orders with less than 1% relative abundance. (B) Venn diagram displaying bacterial orders with regard to different sample types FV: *Fucus vesiculosus*, FS: *F. serratus,* FE*: F. distichus* subsp. *evanescens,* BF: biofilm on stone, SW: seawater.

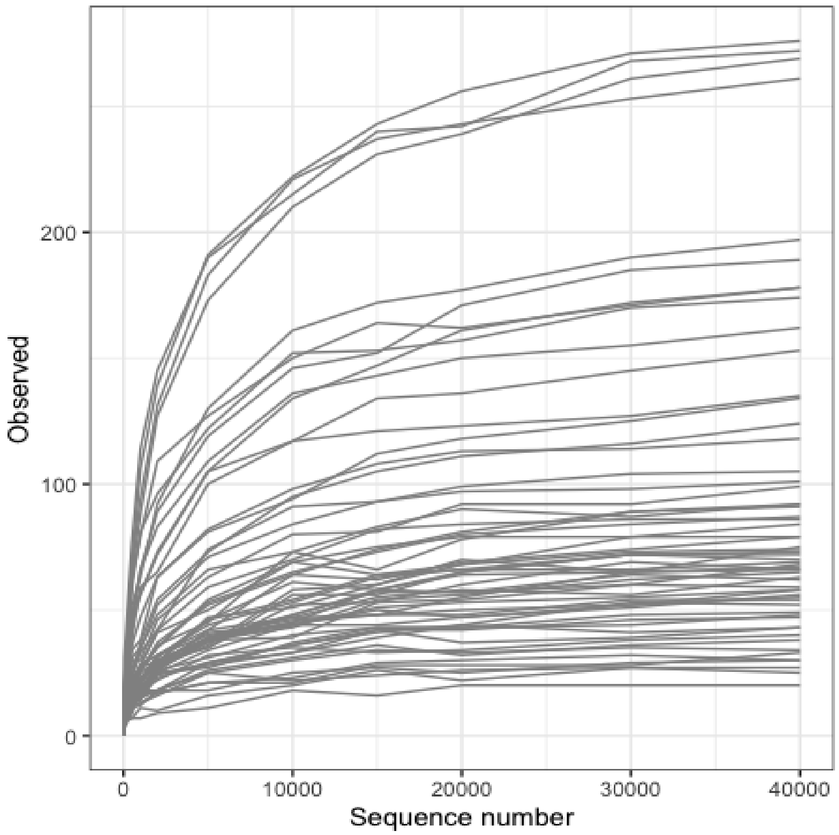

Figure S18. Rarefaction curves of eukaryotic ITS region amplicon sequences from all 60 samples.

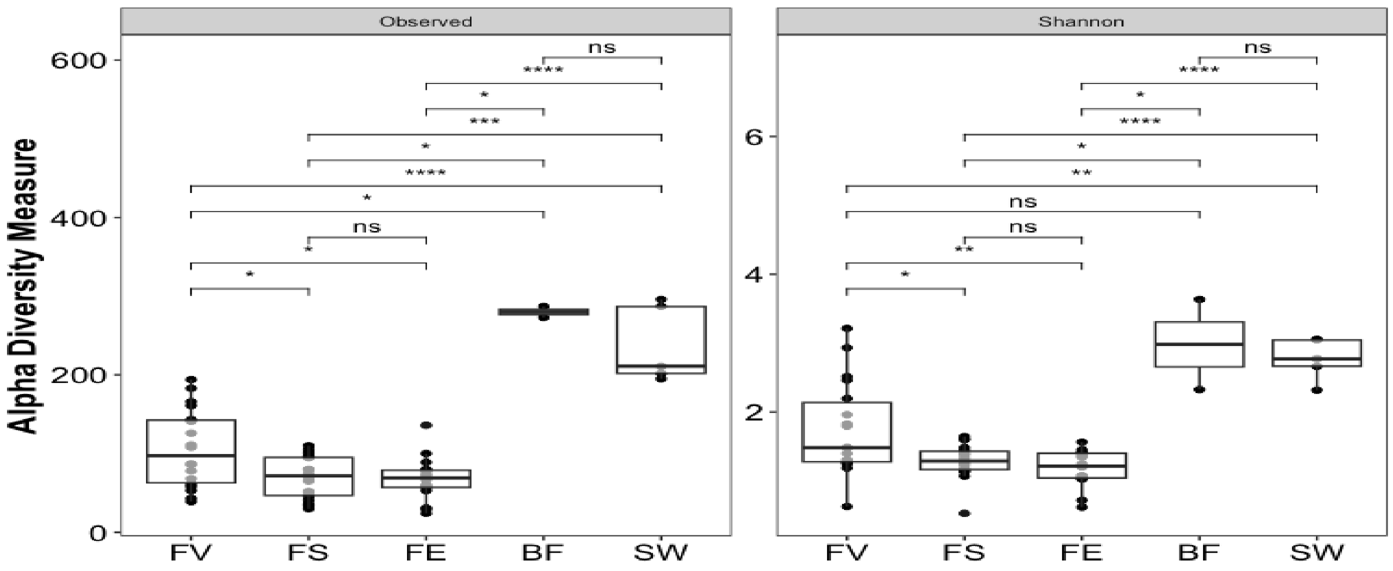

Figure S19. Alpha diversity (ASV-Observed vs. Shannon) of eukaryotic epiphytic community based on ITS fragment sequences with regard to sample source. FV: *F. vesiculosus*, FS: *F. serratus*, FE: *F. distichus* subsp. *evanescens,* BF: biofilm on stone, SW: seawater. Significance levels: >0:****, >0.0001: ***, >0.001: **, 0.01: *, >0.05: ns)

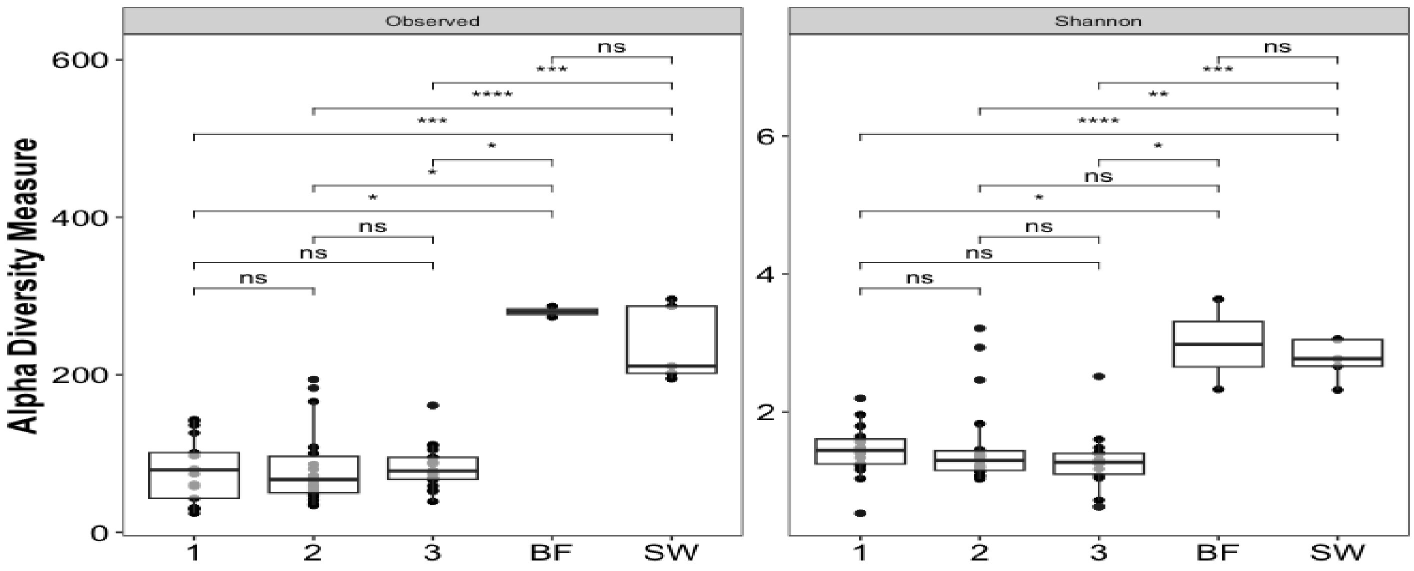

Figure S20. Alpha diversity (ASV-Observed vs. Shannon) of eukaryotic epiphytic community based on ITS fragment sequences with regard to individual. 1: individual 1, 2: individual 2, 3: individual 3, BF: biofilm on stone, SW: seawater. Significance levels: >0:****, >0.0001: ***, >0.001: **, 0.01: *, >0.05: ns)

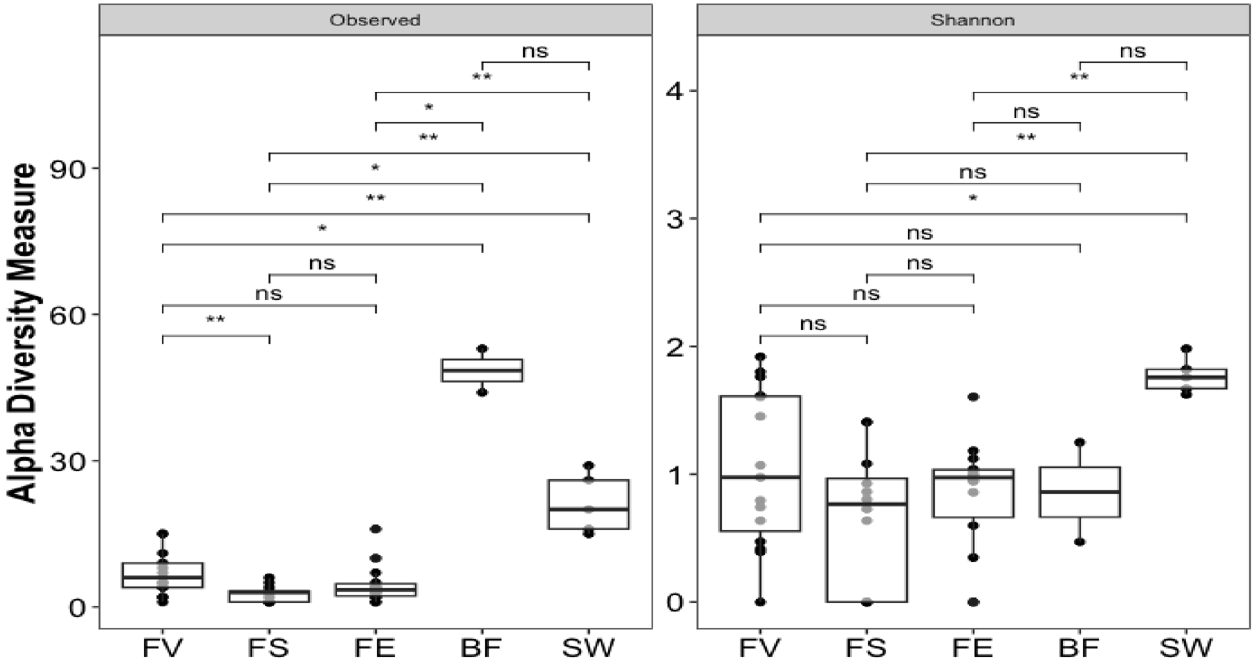

Figure S21. Alpha diversity (ASV-Observed vs. Shannon) of fungal epiphytic community based on ITS fragment sequences with regard to sample source. FV: *F. vesiculosus*, FS: *F. serratus*, FE: *F. distichus* subsp. *evanescens,* BF: biofilm on stone, SW: seawater. Significance levels: >0:****, >0.0001: ***, >0.001: **, 0.01: *, >0.05: ns)

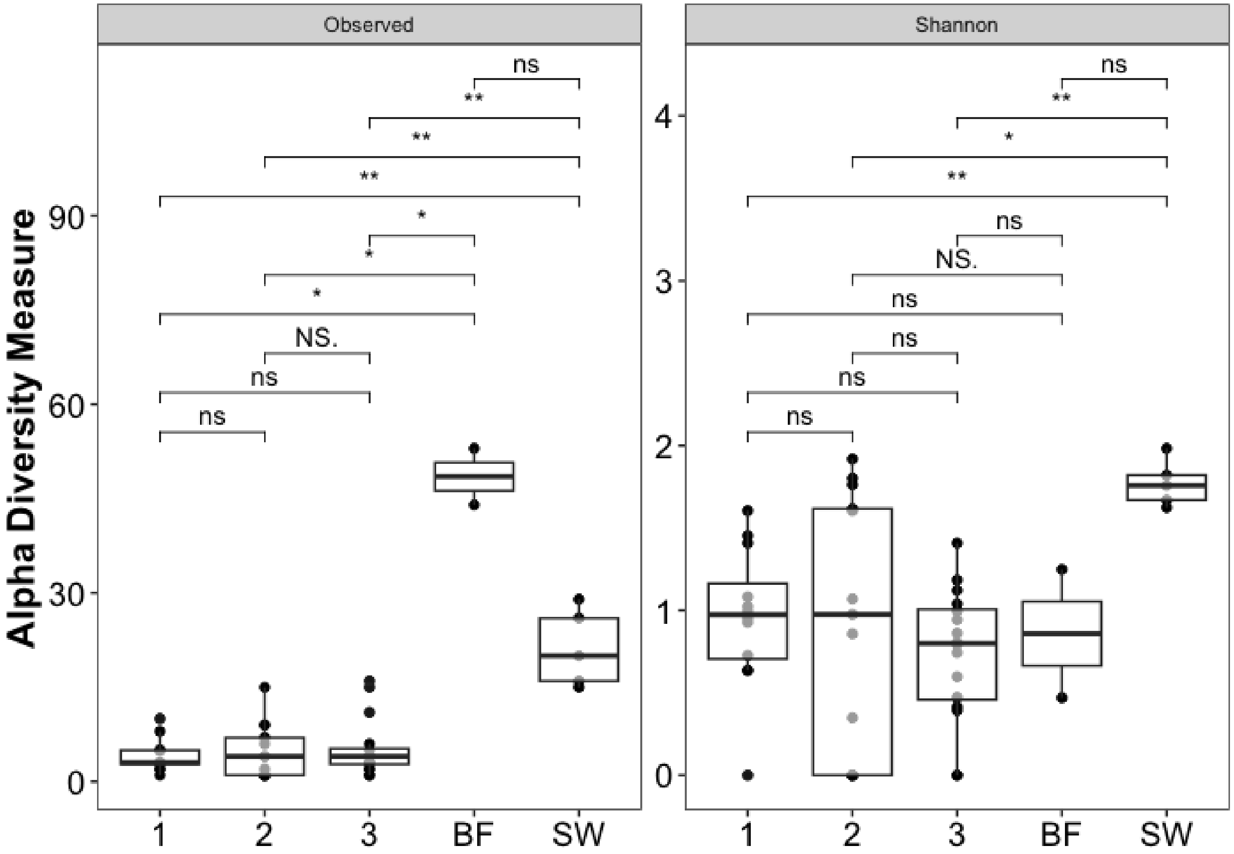

Figure S22. Alpha diversity (ASV-Observed vs. Shannon) of fungal epiphytic community based on ITS fragment sequences with regard to individual. 1: individual 1, 2: individual 2, 3: individual 3, BF: biofilm on stone, SW: seawater. Significance levels: >0:****, >0.0001: ***, >0.001: **, 0.01: *, >0.05: ns)

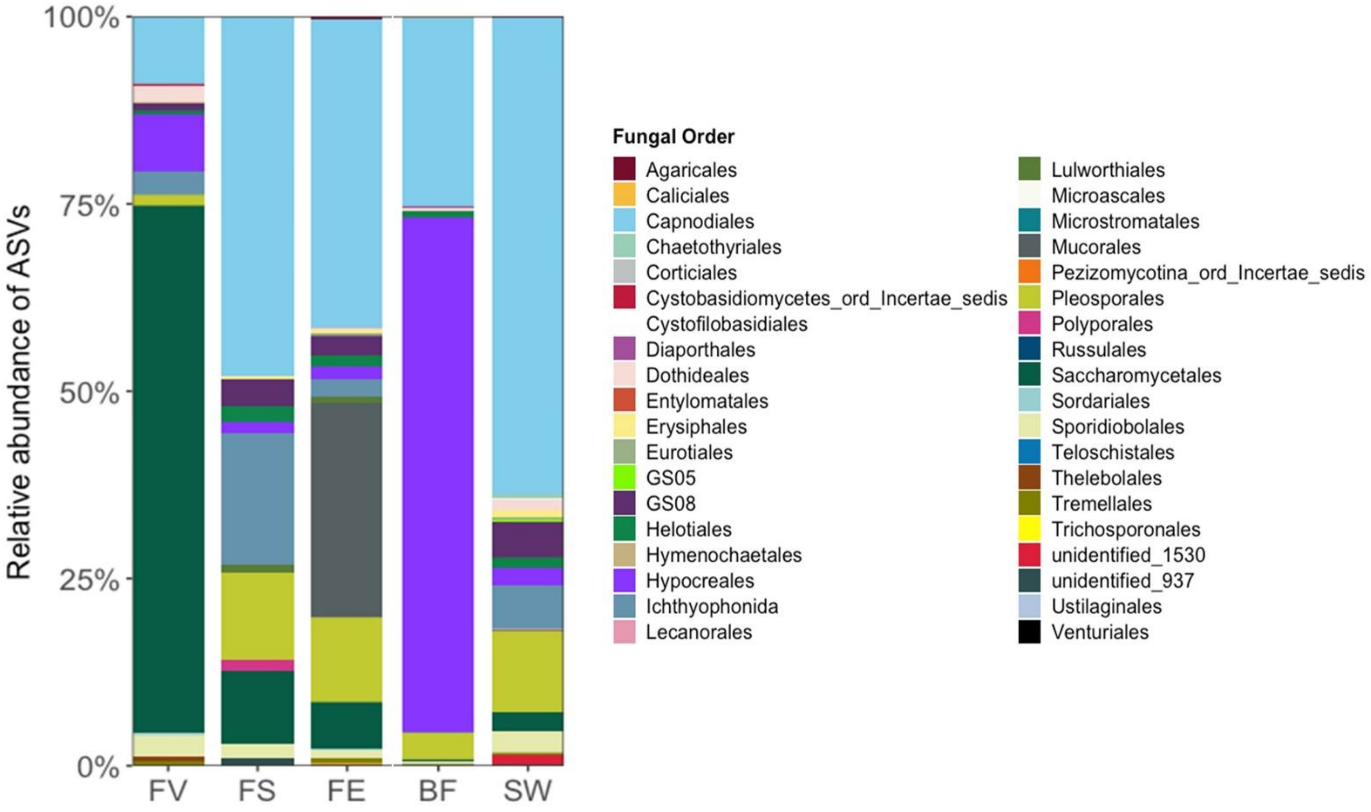

Figure S23. Fungal orders associated to surfaces of *Fucus* spp., and stone biofilm (BF) and seawater (SW) reference samples. FV: *Fucus vesiculosus*, FS: *F. serratus*, FE: *F. distichus* subsp. *evanescens*.

Table S2

Relative abundances of bacterial genera (> 1%) associated to surfaces of *Fucus* spp., seawater and stone biofilm based on amplicon sequencing of the V3/V4 region of the 16S rRNA gene.

F. vesiculosus (FV)

ASV No.	Genus	Phylum	% Abun-dance
ASV1	Schizothrix LEGE 07164	Cyanobacteria	24.41
ASV11	Sva0996 marine group	Actinobacteriota	11.78
ASV12	Ilumatobacter	Actinobacteriota	9.85
ASV10	Yoonia-Loktanella	Proteobacteria	8.68
ASV20	Octadecabacter	Proteobacteria	6.85
ASV17	Litorimonas	Proteobacteria	5.08
ASV14	Silicimonas	Proteobacteria	5.00
ASV51	Erythrobacter	Proteobacteria	4.08
ASV4	Robiginitomaculum	Proteobacteria	2.74
ASV39	Filomicrobium	Proteobacteria	2.48
ASV13	Candidatus Tenderia	Proteobacteria	2.04
ASV73	Altererythrobacter	Proteobacteria	1.54
ASV21	Fretibacter	Proteobacteria	1.35
ASV61	Phormidesmis ANT.LACV5.1	Cyanobacteria	1.24
ASV24	Truepera	Deinococcota	1.09

F. serratus (FS)

ASV No.	Genus	Phylum	% Abun-dance
ASV1	Schizothrix LEGE 07164	Cyanobacteria	29.61
ASV11	Sva0996 marine group	Actinobacteriota	13.97
ASV17	Litorimonas	Proteobacteria	13.65
ASV12	Ilumatobacter	Actinobacteriota	8.31
ASV24	Truepera	Deinococcota	6.24
ASV4	Robiginitomaculum	Proteobacteria	3.62
ASV21	Fretibacter	Proteobacteria	3.50
ASV10	Yoonia-Loktanella	Proteobacteria	2.99
ASV68	Blastopirellula	Planctomycetota	2.67
ASV20	Octadecabacter	Proteobacteria	2.36
ASV88	Acaryochloris MBIC11017	Cyanobacteria	1.71
ASV14	Silicimonas	Proteobacteria	1.59
ASV61	Phormidesmis ANT.LACV5.1	Cyanobacteria	1.39
ASV39	Filomicrobium	Proteobacteria	1.05

F. distichus subsp. evanescens (FE)

ASV No.	Genus	Phylum	% Abun-dance
ASV1	Schizothrix LEGE 07164	Cyanobacteria	26.02
ASV4	Robiginitomaculum	Proteobacteria	12.84
ASV10	Yoonia-Loktanella	Proteobacteria	8.96
ASV11	Sva0996 marine group	Actinobacteriota	7.20
ASV12	Ilumatobacter	Actinobacteriota	7.12
ASV13	Candidatus Tenderia	Proteobacteria	5.25
ASV20	Octadecabacter	Proteobacteria	3.96
ASV17	Litorimonas	Proteobacteria	3.86
ASV68	Blastopirellula	Planctomycetota	2.90
ASV21	Fretibacter	Proteobacteria	2.69
ASV19	Clade Ia	Proteobacteria	2.03
ASV14	Silicimonas	Proteobacteria	1.75
ASV61	Phormidesmis ANT.LACV5.1	Cyanobacteria	1.64
ASV29	Planktomarina	Proteobacteria	1.31

seawater (SW)

ASV No.	Genus	Phylum	% Abun-dance
ASV19	Clade Ia	Proteobacteria	22.46
ASV1	Schizothrix LEGE 07164	Cyanobacteria	14.56
ASV29	Planktomarina	Proteobacteria	10.17
ASV79	Cand. Puniceispirillum	Proteobacteria	6.02
ASV12	Ilumatobacter	Actinobacteriota	4.89
ASV11	Sva0996 marine group	Actinobacteriota	4.13
ASV4	Robiginitomaculum	Proteobacteria	4.09
ASV130	Aphanizomenon NIES81	Cyanobacteria	3.44
ASV158	Cyanobium PCC-6307	Cyanobacteria	3.42
ASV10	Yoonia-Loktanella	Proteobacteria	3.38
ASV171	Propionigenium	Fusobacteriota	2.09
ASV17	Litorimonas	Proteobacteria	1.88
ASV20	Octadecabacter	Proteobacteria	1.73
ASV61	Phormidesmis ANT.LACV5.1	Cyanobacteria	1.52
ASV14	Silicimonas	Proteobacteria	1.28

stone biofilm (BF)

ASV No.	Genus	Phylum	% Abun-dance
ASV61	Phormidesmis ANT.LACV5.1	Cyanobacteria	12.76
ASV49	Rivularia PCC-7116	Cyanobacteria	11.30
ASV12	Ilumatobacter	Actinobacteriota	9.39
ASV1	Schizothrix LEGE 07164	Cyanobacteria	8.85
ASV13	Cand. Tenderia	Proteobacteria	5.80
ASV10	Yoonia-Loktanella	Proteobacteria	5.07
ASV76	Sphingorhabdus	Proteobacteria	4.66
ASV116	Pleurocapsa PCC-7319	Cyanobacteria	4.37
ASV93	Phormidium MBIC10003	Cyanobacteria	4.05
ASV11	Sva0996 marine group	Actinobacteriota	3.06
ASV51	Erythrobacter	Proteobacteria	1.89
ASV98	Ahrensia	Proteobacteria	1.61
ASV4	Robiginitomaculum	Proteobacteria	1.54
ASV14	Silicimonas	Proteobacteria	1.34
ASV19	Clade Ia	Proteobacteria	1.25
ASV158	Cyanobium PCC-6307	Cyanobacteria	1.22
ASV29	Planktomarina	Proteobacteria	1.12
ASV20	Octadecabacter	Proteobacteria	1.10

Table S3

Bacterial beta diversity statistics based on Bray-Curtis dissimilarity, left: PERMANOVA results, right: pairwise permutation test for homogeneity of multivariate dispersions

**PERMANOVA**	Signif. codes: 0 '***' 0.001 '**' 0.01 '*' 0.05 '.' 0.1 ' ' 1								
	Df	Sum Squares	R2	F	Pr(>F)	Signifi-cance	Resi-duals df	Residuals Sum Squares	Residuals R2
**full dataset**									
origin	4	4.8181	0.39588	9.6655	0.001	***	59	7.3572	0.60412
indivdual no.	4	3.1344	0.25753	5.1162	0.001	***	59	9.0364	0.74247
**FV**									
indivdual no.	2	0.50452	0.26094	2.6481	0.015	*	15	1.42895	0.73906
tissue age	1	0.58970	0.30499	7.0214	0.001	***	16	1.3438	0.69501
**FS**									
indivdual no.	2	1.16040	0.42611	5.1974	0.001	***	14	1.5629	0.57389
**FE**									
indivdual no.	2	0.39425	0.23931	2.2022	0.013	*	14	1.25319	0.76069

**pairwise permutation for homogeneity of multivariate dispersion**										
	Df	Sum Squares	Mean Squares	F	N permut	Pr(>F)	Signifi-cance	Resi-duals df	Residuals sum squares	Residuals mean squares
**full dataset**										
origin	4	0.13976	0.034941	8.1559	1000	0.000999	***	59	0.25276	0.004284
indivdual no.	4	0.13753	0.034383	7.4047	1000	0.000999	***	59	0.27396	0.004643
**FV**										
indivdual no.	2	0.00199	0.000966	0.2325	1000	0.7972	ns	15	0.064251	0.004283
tissue age	1	0.00586	0.005862	1.361	1000	0.2677	ns	16	0.068909	0.004307
**FS**										
indivdual no.	2	0.03316	0.01658	2.1023	1000	0.1638	ns	14	0.110414	0.007887
**FE**										
indivdual no.	2	0.00709	0.003544	1.0939	1000	0.3716	ns	14	0.045354	0.00324

Table S4

Relative abundances of eukaryote genera (> 1%) associated to surfaces of *Fucus* spp., seawater and stone biofilm based on amplicon sequencing of the ITS fragment.

F. vesiculosus (FV)			
**ASV No.**	**Genus**	**Phylum**	**% Abundance**
ASV52	Zoothamnium	Ciliophora	39.26
ASV45	unidentified_91524	Ciliophora	28.84
ASV64	Candida	Ascomycota	16.26
ASV106	Pseudovorticella	Ciliophora	3.83
ASV178	unidentified_15	unidentified	2.32
ASV210	Myoschiston	Ciliophora	1.92
ASV10	Haptocillium	Ascomycota	1.82
ASV216	unidentified_4835	unidentified_6334	1.62

F. serratus (FS)			
**ASV No.**	**Genus**	**Phylum**	**% Abundance**
ASV45	unidentified_91524	Ciliophora	33.17
ASV87	Ulva	Chlorophyta	17.18
ASV19	unidentified_12446	Ciliophora	11.57
ASV106	Pseudovorticella	Ciliophora	9.69
ASV28	Sarsia	Cnidaria	7.14
ASV280	Uronema_91786	Ciliophora	5.94
ASV108	unidentified_5383	unidentified_6967	4.17
ASV366	Haliclystus	Cnidaria	2.07
ASV52	Zoothamnium	Ciliophora	1.25

F. distichus subsp. *evanescens* (FE)			
**ASV No.**	**Genus**	**Phylum**	**% Abundance**
ASV28	Sarsia	Cnidaria	27.22
ASV19	unidentified_12446	Ciliophora	25.46
ASV221	Mucor	Mucoromycota	11.60
ASV87	Ulva	Chlorophyta	8.74
ASV45	unidentified_91524	Ciliophora	6.17
ASV108	unidentified_5383	unidentified_6967	3.81
ASV97	Alternaria	Ascomycota	2.64
ASV127	Pseudendoclonium	Chlorophyta	2.22
ASV106	Pseudovorticella	Ciliophora	2.08
ASV166	unidentified_33279	Chlorophyta	1.23
ASV244	unidentified_2488	Rozellomycota	1.02

seawater (SW)			
**ASV No.**	**Genus**	**Phylum**	**% Abundance**
ASV28	Sarsia	Cnidaria	39.62
ASV19	unidentified_12446	Ciliophora	35.05
ASV108	unidentified_5383	unidentified_6967	5.81
ASV45	unidentified_91524	Ciliophora	4.89
ASV87	Ulva	Chlorophyta	3.30
ASV106	Pseudovorticella	Ciliophora	1.94

stone biofilm (BF)			
**ASV No.**	**Genus**	**Phylum**	**% Abundance**
ASV10	Haptocillium	Ascomycota	76.14
ASV87	Ulva	Chlorophyta	5.84
ASV75	Cladosporium	Ascomycota	3.94
ASV127	Pseudendoclonium	Chlorophyta	3.47
ASV97	Alternaria	Ascomycota	2.80
ASV45	unidentified_91524	Ciliophora	2.34
ASV166	unidentified_33279	Chlorophyta	1.17

Table S5

ITS beta diversity statistics based on Bray-Curtis dissimilarity, left: PERMANOVA results, right: pairwise permutation test for homogeneity of multivariate dispersions

**PERMANOVA**		Signif. codes: 0 ‘***’ 0.001 ‘**’ 0.01 ‘*’ 0.05 ‘.’ 0.1 ‘ ’ 1								**pairwise permutation for homogeneity of multivariate dispersion**									
	Df	Sum Squares	R2	F	Pr(>F)	Signifi-cance	Resi-duals df	Residuals Sum Squares	Residuals R2	Df	Sum Squares	Mean Squares	F	N permut	Pr(>F)	Signifi-cance	Resi-duals df	Residuals sum squares	Residuals mean squares
**full dataset**										**full dataset**									
origin	4	4.0572	0.29338	5.7089	0.001	***	55	9.7718	0.70662	4	0.13976	0.034941	8.1559	1000	0.000999	***	59	0.25276	0.004284
indivdual no.	4	3.7628	0.2721	5.1398	0.001	***	55	10.0662	0.7279	4	0.13753	0.034383	7.4047	1000	0.000999	***	59	0.27396	0.004643
**FV**										**FV**									
indivdual no.	2	0.7918	0.2223	2.1438	0.054	.	15	2.77	0.7777	2	0.00199	0.000966	0.2325	1000	0.7972	ns	15	0.064251	0.004283
**FS**										**FS**									
indivdual no.	2	0.37763	0.18583	1.7118	0.076	.	15	1.65453	0.81417	2	0.03316	0.01658	2.1023	1000	0.1638	ns	14	0.110414	0.007887
**FE**										**FE**									
indivdual no.	2	0.27035	0.09155	0.7054	0.493	ns	14	2.68281	0.90845	2	0.00709	0.003544	1.0939	1000	0.3716	ns	14	0.045354	0.00324

Table S6

Relative abundances of fungal genera (>1%) associated to the surfaces of *Fucus* spp., seawater and stone biofilm based on amplicon sequencing of the ITS fragment.

F. vesiculosus (FV)			
**ASV No.**	**Genus**	**Phylum**	**% Abun-dance**
ASV64	Candida	Ascomycota	82.30
ASV10	Haptocillium	Ascomycota	9.20
ASV186	Sphaeroforma	Ichthyosporia_phy_Incertae_sedis	3.68
ASV97	Alternaria	Ascomycota	1.61
ASV244	unidentified_2488	Rozellomycota	1.18

F. serratus (FS)			
**ASV No.**	**Genus**	**Phylum**	**% Abun-dance**
ASV186	Sphaeroforma	Ichthyosporia_phy_Incertae_sedis	38.04
ASV97	Alternaria	Ascomycota	25.00
ASV342	Metschnikowia	Ascomycota	17.39
ASV244	unidentified_2488	Rozellomycota	7.61
ASV1291	Wickerhamomyces	Ascomycota	3.26
ASV451	Claviceps	Ascomycota	3.26
ASV1208	unidentified_786	Ascomycota	2.17
ASV871	unidentified_99109	Ascomycota	2.17
ASV1107	Blumeria	Ascomycota	1.09

F. distichus subsp. evanescens (FE)			
**ASV No.**	**Genus**	**Phylum**	**% Abun-dance**
ASV221	Mucor	Mucoromycota	65.41
ASV97	Alternaria	Ascomycota	14.91
ASV244	unidentified_2488	Rozellomycota	5.77
ASV186	Sphaeroforma	Ichthyosporia_phy_Incertae_sedis	5.37
ASV871	unidentified_99109	Ascomycota	2.19

seawater (SW)			
**ASV No.**	**Genus**	**Phylum**	**% Abun-dance**
ASV97	Alternaria	Ascomycota	26.17
ASV186	Sphaeroforma	Ichthyosporia_phy_Incertae_sedis	17.73
ASV244	unidentified_2488	Rozellomycota	14.69
ASV75	Cladosporium	Ascomycota	13.98
ASV342	Metschnikowia	Ascomycota	7.11
ASV451	Claviceps	Ascomycota	4.30
ASV440	unidentified_1163	Chytridiomycota	3.67
ASV573	Pyrenophora	Ascomycota	2.89
ASV568	Knufia	Ascomycota	1.64

stone biofilm (BF)			
**ASV No.**	**Genus**	**Phylum**	**% Abun-dance**
ASV10	Haptocillium	Ascomycota	90.82
ASV75	Cladosporium	Ascomycota	4.70
ASV97	Alternaria	Ascomycota	3.35
